# Predictors of Junior Versus Senior Elite Performance are Opposite: A Systematic Review and Meta-Analysis of Participation Patterns

**DOI:** 10.1007/s40279-021-01625-4

**Published:** 2022-01-17

**Authors:** Michael Barth, Arne Güllich, Brooke N. Macnamara, David Z. Hambrick

**Affiliations:** 1 Department of Business and Society, University of Applied Sciences Kufstein Tyrol, Kufstein, Austria; 2 Department of Sport Science, University of Innsbruck, Innsbruck, Tyrol Austria; 3 Department of Sports Science, University of Technology Kaiserslautern, Kaiserslautern, Germany; 4 Department of Psychological Sciences, Case Western Reserve University, Cleveland, OH USA; 5 Department of Psychology, Michigan State University, East Lansing, MI USA

## Abstract

**Background:**

Does early specialization facilitate later athletic excellence, or is early diversification better? This is a longstanding theoretical controversy in sports science and medicine. Evidence from studies investigating athletes’ starting age, childhood/adolescent progress, and amounts of coach-led practice and peer-led play in their main sport and in other sports has been mixed. Each participation variable was positively correlated with performance in some studies but uncorrelated or negatively correlated with performance in others. However, samples were heterogeneous in age, sports, and performance levels.

**Objective:**

This study aimed to establish robust, generalizable findings through a systematic review and meta-analysis. We investigated three questions: (1) did higher- and lower-performing athletes differ in childhood/adolescent progress, starting age, or amounts of main-sport or other-sports practice or play; (2) do effects differ between junior and adult athletes, compared performance levels, or types of sports; and (3) are effect sizes from different predictors associated with one another?

**Methods:**

We conducted a systematic literature search in SPORTDiscus, ERIC, ProQuest, PsycINFO, PubMed, Scopus, WorldCat, and Google Scholar until 28 February 2021. Selection criteria included original research studies comparing higher- versus lower-performing athletes regarding one or more of our predictor variables within defined age categories, sports, and sex, and reporting effect sizes or data needed to compute effects sizes. Mean meta-analytic Cohen’s *d* was calculated for each predictor. Quality of evidence was evaluated using GRADE.

**Results:**

In total, 71 study reports met all eligibility criteria and included 262 international athlete samples, 685 effect sizes, and a total sample size of 9241 athletes from local to Olympic competition level and from diverse sports. The following findings emerged. (1) Compared with their national-class counterparts, adult world-class athletes had more childhood/adolescent multi-sport coach-led practice, a later main-sport start, less main-sport practice, and slower initial progress (|0.23|< $\bar{d }$<|0.50|; all *p* < 0.001). (2) The opposite was true for predictors of junior-age performance: higher-performing juniors had an earlier main-sport start, more main-sport practice, less other-sports practice, and faster initial progress (|0.23|< $\bar{d }$< |0.61|; all *p* < 0.001). (3) Main-sport or other-sports peer-led play had negligible effects (all *p* > 0.05). (4) Results were robust across types of sports. (5) Effect sizes from different predictors were associated with one another (|0.64|< *r* <|0.79|). A GRADE assessment revealed a low quality of evidence for peer-led play but a moderate to high quality of evidence for all other predictors.

**Discussion:**

Excess childhood/adolescent specialized practice may hinder athletes’ long-term development through overuse injury, burnout, suboptimal athlete–sport match, and limiting long-term learning capital. By contrast, adult world-class athletes’ childhood/adolescent multi-sport practice with reduced main-sport practice implied a relatively resource-preserving, cost-reducing, and risk-buffering pattern that yielded greater long-term sustainability and practice efficiency.

**Supplementary Information:**

The online version contains supplementary material available at 10.1007/s40279-021-01625-4.

## Key Points

**Table:** 

Short-term junior-age athletic success is facilitated by an early start in the main sport, rapid initial progress, and intensive specialized coach-led practice in the main sport, with little or no practice in other sports.
Long-term adult-age success is facilitated by extensive childhood/adolescent multi-sport practice, relatively late start in the main sport, gradual initial progress, and only moderate childhood/adolescent specialized main-sport practice.
Peer-led play in the main sport or in other sports has negligible effects on both junior and senior performance.

## Introduction

Does early specialization facilitate later athletic excellence? Or is early diversification with multi-sport practice and play better? This is a longstanding theoretical controversy in sports science and medicine [1–7]. Although there is consensus that extensive experience over multiple years is required to develop exceptional performance, the optimum type and amount of developmental sport activities is subject to ongoing debate. The patterns of early specialization and early diversification are implied in the most popular (i.e., most-cited) frameworks of talent development in sports science literature, the deliberate practice view [8]; with special reference to sports [9, 10] and the Developmental Model of Sport Participation (DMSP) [11]. For recent identical reviews, see Erickson et al. [12], Côté and Erickson [13], and Côté et al. [14].

Ericsson et al. [8] proposed that performance is monotonically related to the cumulative amount of deliberate practice: task-specific practice under the supervision and monitoring of a coach (i.e., coach-led practice) that is undertaken to improve performance, is highly effortful, and is not inherently enjoyable. Ericsson et al. [8] asserted that deliberate practice is the most effective type of activity to improve performance and so proposed an early start and subsequent maximization of deliberate practice. By inference, investing time and effort in other types of sport activities—practice in other sports or play activities in the main sport or in other sports—reduces the amount of deliberate practice and thereby performance. Ericsson et al. [8] also emphasized the importance of rapid initial performance progress (for similar views from a giftedness perspective, see Hohmann and Seidel [15], Heller et al. [16], and Gagné [17]).

In contrast, the early diversification path of the DMSP [11] holds that, although deliberate practice is necessary, single-sport specialization and intensive deliberate practice should not commence until adolescence. This late specialization should be preceded by extensive childhood/adolescent deliberate play in various sports: “pick-up” games that are regulated by the participants, not by a coach (i.e., peer-led play), and are undertaken for the inherent enjoyment of the game rather than to improve performance (e.g., pick-up soccer, basketball, table tennis in schoolyards, playgrounds, parks, and streets).

Early specialization and early diversification have typically been regarded as two contrasting, dichotomous participation patterns [1, 3, 4, 11, 18, 19], but this is an imprecise characterization. An athlete’s participation pattern is generally characterized by several continuous, quantitative variables, including starting age and amounts of coach-led practice and peer-led play, both in the athlete’s main sport and in other sports [20]. These continuous variables thus provide a more accurate and detailed description of athletes’ participation patterns. In addition, to investigate relationships between these participation variables and performance, an artificially dichotomized specialization–diversification construct is neither needed nor beneficial. Therefore, we do not follow the dichotomized specialization–diversification approach, but rather focus on continuous, quantitative participation variables.

The empirical evidence from studies using these continuous predictor variables is mixed [21–23]. Each of the participation variables has been found to be positively correlated with performance in some studies but uncorrelated or negatively correlated with performance in others. However, when distinguishing studies based on the performance levels compared and whether the samples were junior (youth) or senior (adults competing in the open-age category, typically in their 20s and 30s) athletes, some consistency became apparent [22, 23]. In numerous studies, higher junior performance was correlated with a faster rate of childhood/adolescent performance progress, greater amounts of main-sport coach-led practice, and less other-sports practice. By contrast, studies comparing the highest adult performance levels—senior world class and national class—suggested that world-class performance was associated with greater amounts of (earlier, childhood/adolescent) other-sports coach-led practice and slower childhood/adolescent progress and was uncorrelated or negatively correlated with the amount of main-sport coach-led practice.

Among empirical studies examining predictors of sports performance, an analysis of the samples revealed wide ranges of age categories (juniors, seniors), types of sports, and performance levels (local to Olympic competition level). Furthermore, past studies typically analysed effects of predictor variables separately and did not consider potential associations between effects of different predictors, with a few exceptions [24–28].

### Present Study

The present meta-analysis aimed to establish more robust and generalizable findings by synthesizing the results from 71 study reports, comprising 685 effect sizes from 9241 athletes. Many of the data sets, particularly those including adult world-class athletes, have only become available recently (especially since 2016). The present sample includes 219 Olympic, world, and continental champions; 474 medalists; and 812 athletes achieving top-ten placings at the major open-age international championships.

Considering the entire range of variables describing the specialization–diversification continuum, we investigated the following three questions:

1. Did higher- and lower-performing athletes differ in childhood/adolescent performance progress, starting age, or amounts of coach-led practice or peer-led play, in either their main sport or in other sports?
2. Do effects of predictor variables differ across athletes’ age category (junior vs. senior), compared performance levels, or types of sports?
3. Are effect sizes from different predictor variables independent or associated with one another?

The deliberate practice view predicts that higher-performing athletes, compared with their lower-performing counterparts, had faster initial performance progress, started their main sport earlier, and accumulated greater amounts of task-specific main-sport coach-led practice through their career (but not greater amounts of any other type of sport activity). In contrast, the diversification path of the DMSP predicts that an athlete’s eventual performance is associated with the amounts of childhood/adolescent multi-sport peer-led play.

Alternatively, based on the available empirical evidence, we hypothesized that predictors of short-term junior performance and of long-term senior performance differ: Among junior performance levels, we expected that higher performance would be positively correlated with rapid childhood/adolescent progress and large amounts of main-sport practice but negatively correlated with other-sports practice. In contrast, among the highest senior performance levels—world class and national class—we expected that a higher eventual performance level would be correlated with relatively slower initial progress and greater amounts of (childhood/adolescent) multi-sport practice but uncorrelated or negatively correlated with amounts of main-sport practice.

## Methods

**Fig. 1:**
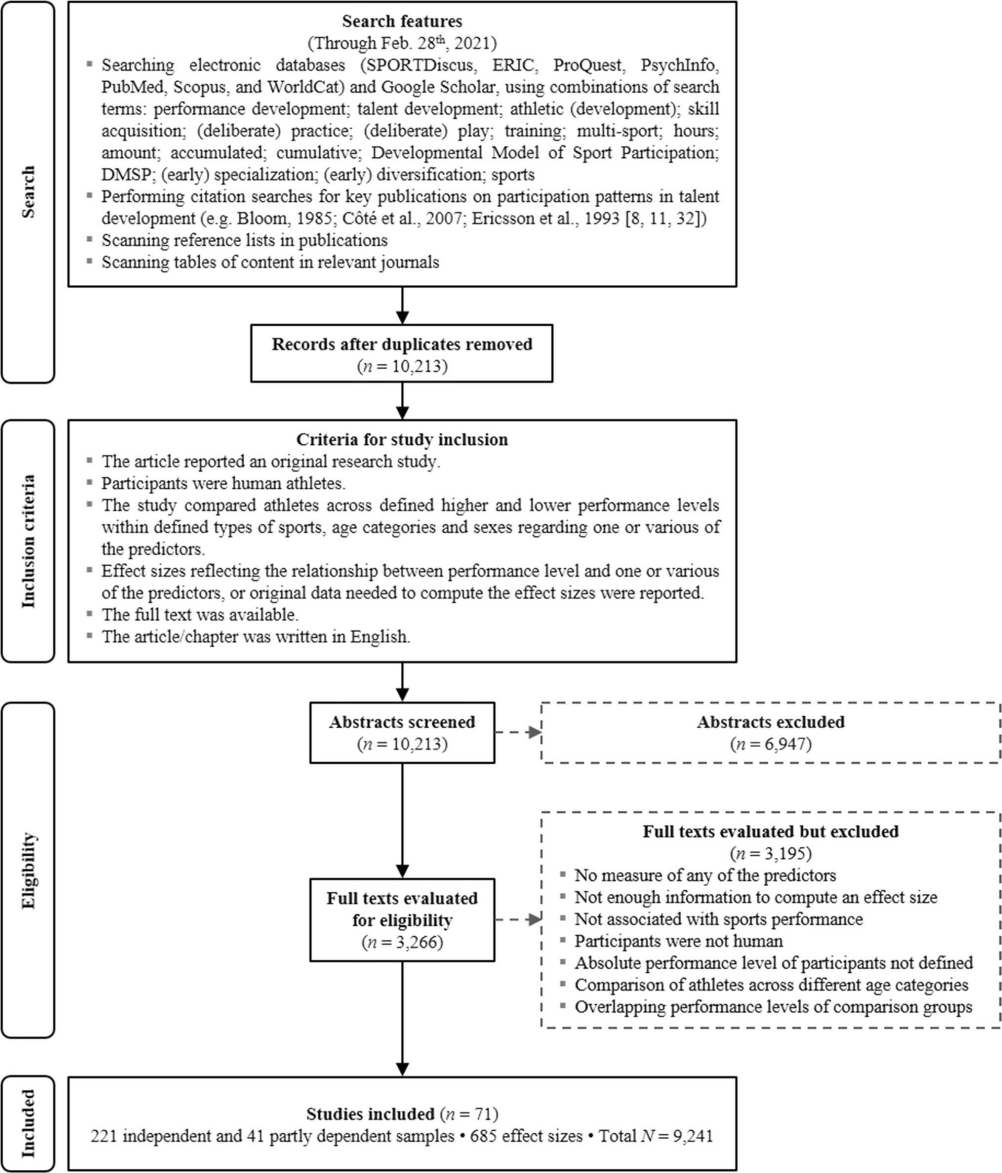
Flow diagram of the literature search and study coding

The study search and selection procedure was guided by the PRISMA (Preferred Reporting Items for Systematic Reviews and Meta-Analyses) statement [29]. We integrated the literature search of Macnamara et al. [21] (search through 14 October 2014) and Güllich et al. [30] (search through 27 February 2019) and updated the literature search through 28 February 2021. This resulted in an additional 20 new study reports, 208 effect sizes, and 3145 athletes. Figure 1 shows the flowchart of the major steps of the combined searches and screenings. We also contacted the authors of 19 study reports by email or phone to request original data or clarifications regarding the competition level of participants or their research methods; all responded (see Table S1 in the electronic supplementary material [ESM]). For one study [31], we excluded the three junior swimmers in the otherwise senior swimmer sample.

### Sample

The search resulted in a total of 71 relevant study reports from 1998 to 2020 (see Table S1 in the ESM for the reference list and a description of each study). Each study was coded for publication status, descriptive data, sample characteristics, research methods, performance levels compared, predictor variables, and effects on performance differences. The study reports included 221 independent and 41 partly dependent athlete samples, with 685 effect sizes and a total sample size of 9241 athletes. Of the 685 effect sizes, 528 were from published articles and 157 from unpublished study reports (theses, manuscripts). As we did not directly interact with participants, institutional ethics approval was not applicable.

Athletes were from North America (9%), Central/South America (2%), Europe (78%), Africa (0.2%), Asia (6%), and Oceania (5%); 67% were male and 33% female. Participants were from a wide range of sports: 24% from “cgs” sports (where performance is measured in centimeters, grams, or seconds, e.g., athletics, rowing, swimming, weightlifting), 67% from game sports (e.g., basketball, soccer, tennis), 2% from combat sports (e.g., fencing, judo, wrestling), 6% from artistic composition sports (e.g., artistic gymnastics, figure skating, platform diving), and 2% from other types of sports (meeting none or multiple criteria of the aforementioned sports types, e.g., modern pentathlon, ski jumping). Two studies involved Paralympic athletes. Within junior game-sport samples, male soccer players were over-represented (65%).

We distinguished between junior and senior athletes. This distinction is important because the populations of successful junior athletes and successful senior athletes are not identical and are largely distinct populations: most successful juniors do not become successful senior athletes, whereas most successful senior athletes did not achieve equivalent success levels in former junior competitions [33, 34]. Junior and senior samples were defined based on the definitions of the junior age limit of the international federation for each sport (e.g., female swimming, 17 years; baseball, 18 years; athletics, 19 years). The sample included 5690 junior (62%) and 3551 senior athletes (38%).

We coded the absolute performance level of the samples compared in each study based on their competition levels—world class, national class, regional class, and below. Table 1 defines the performance levels and describes the numbers of participants at each performance level.

**Table 1:** Definition of the performance levels of the participants

Performance level	Definition	Number of athletes		
		Junior	Senior	Total
World class	Athletes won medals and/or placed in the top ten at major international open-age championships (seniors: Olympic games, world, continental championships, Pan American, Commonwealth games), or junior world or continental championships (juniors)	191	812	1003
National class	Athletes were members of the national selection team or squad and/or placed in the top ten at national championships and/or played in the highest national league (but did not place in the top ten at major international championships)	3322	1496	4818
Regional class	Athletes competed below the national and above the county level (e.g., 2nd-tier, 3rd-tier, etc. league; NCAA conferences; minor league baseball; state or provincial level championships and leagues)	1777	764	2541
Below	Athletes competed at a local or up to county level	400	479	879
Total		5690	3551	9241

*NCAA* National Collegiate Athletic Association

Most athletes were from industrialized countries (Australia, Canada, Japan, the USA, and Western European countries) and played sports in which their country is internationally competitive. Likewise, athletes from “developing” countries typically played sports in which their country is internationally competitive (e.g., Brazilian volleyball, Kenyan long-distance running, Malaysian badminton).

Based on the absolute performance levels of the samples, we assessed the relative distance between performance levels compared within each study. Comparisons of higher- and lower-performing athletes within a competition level (e.g., within first league players, within state championship participants), among full professionals in the highly professionalized sports (e.g., soccer in Europe, baseball and basketball in the USA), or among neighboring competition levels (e.g., international vs. national championship participants, state vs. provincial championships) were defined as a “narrow bandwidth.” Comparisons across two competition levels were defined as a “medium bandwidth” (e.g., international vs. state level). Wider performance differences were defined as “extreme contrast” comparisons (e.g., world class vs. provincial or lower level). The question of what distinguishes the most outstanding performers from those just below—i.e., senior world-class vs. national-class athletes—is critical from both a theoretical perspective and a practical perspective. On the other hand, “extreme contrast” comparisons (e.g., Olympic medalists vs. local-level competitors) are less relevant from both a theoretical and a practical perspective. In addition, they apply the weakest form of hypothesis testing and favor type I error (for example, see Ackerman [35]).

Athlete performance level and performance differences were determined in the original studies by athlete competition level (*N* = 5634), coaches’ performance assessments within a defined competition level (*N* = 3379; this included coaches’ nomination of athletes for selection teams/squads), and—in three studies—measurement of representative perceptual–motor skills in the laboratory within a defined competition level (*N* = 228).

To measure athletes’ developmental milestones and sport activities through their entire career, oftentimes spanning ≥ 10 years, the methods of choice are retrospective sport-biographic interviews or questionnaires. Interviews were used for 761 athletes and questionnaires for 8115 athletes. Three studies did not define whether data were collected by interviews or questionnaires (*N* = 289), and one study determined athlete age of entering a youth sport academy by document analysis (*N* = 76). Of the total 685 effect sizes, 643 were based on group comparisons (*N* = 8343) and 42 on correlation analyses (*N* = 898). Effect sizes did not differ by the various approaches used in the studies to determine performance differences (0.004 < *F* < 1.467; 0.256 < *p* < 0.953), methods of data collection (0.938 < *F* < 1.901; 0.177 < *p* < 0.341), or data analysis (0.064 < *F* < 1.202; 0.280 < *p* < 0.801) (overall or within age categories for *k* ≥ 5 [36]).

### Predictor Variables

We investigated the effects of the following factors on individual differences in eventual performance:

- Age to reach defined performance-related “milestones” (age to reach milestones).
- Age of starting engagement in the athlete’s main sport (starting age).
- Accumulated amount of coach-led practice in the athlete’s main sport (main-sport practice).
- Accumulated amount of peer-led play in the athlete’s main sport (main-sport play).
- Accumulated amount of coach-led practice in sports other than the athlete’s main sport (other-sports practice).
- Accumulated amount of peer-led play in other sports (other-sports play).

Several studies did not distinguish the amounts of practice and play in other sports, instead pooling them. We therefore formed an additional predictor variable pooling the effects of practice and/or play in other sports (other-sports practice/play). Table 2 describes the predictor variables and defines the empirical indicators used in the original study reports. Given that the theoretical concepts of talent development (deliberate practice, DMSP) particularly differ in proposed childhood/adolescent participation patterns we considered both the amount of a sport activity accumulated through an athlete’s entire athletic career and the early amount accumulated up to age 15 years.

**Table 2:** Empirical indicators of the predictor variables

Predictor variable	Empirical indicators in study reports
Age-related predictors	
Age to reach milestones	The age at which the athlete achieved defined performance-related developmental milestones, including first participation in a national championship, in an international championship; first nomination for a federation’s selection team/squad; and years to the athlete’s present career peak performance
Starting age	The age at which the athlete started engaging in practice and/or competitions in their respective main sport
Amount of different types of sport activity	
Main-sport practice	Accumulated practice amount (number of sessions and/or hours) through the athletic career and/or through defined age categories
Main-sport play, other-sports practice, and other-sports play^a^	Whether or not an athlete engaged in an activity type and, if yes, years of engagement, accumulated amount (number of sessions and/or hours) through the athletic career and/or through defined age categories; for engagement in other sports also the number of sports

^a^Across samples, 48–100% of athletes participated in various sports in childhood and/or adolescence. Of these, 69–88% also competed in those sports

### Effect Sizes and Coding

For each meta-analytic model, we used the standardized mean difference (meta-analytic mean Cohen’s $\bar{d }$) between higher- and lower-performing athletes within a type of sport, age category, and sex. The 42 effect sizes based on correlation analyses were converted to Cohen’s *d*. Effects were weighted by the inverse within-study error variance of *d* of each study [37, 38]. Effect sizes ($\bar{d }$ values) of ~ 0.20, ~ 0.50, and ~ 0.80 were considered as small, medium, and large effects, respectively [39].

When a study reported various indicators of one predictor variable, the effect sizes for that study and variable were pooled (e.g., numbers of practice sessions and hours, practice amount at ≤10, 11–12, 13–14, … 21–22 years). Dependent samples were adjusted using Cheung and Chan’s method [40]. Table 3 shows the number of effect sizes and sample sizes for each meta-analytic model.

**Table 3:** Numbers of effect sizes and sample size of the meta-analytic models

Predictor variable	Junior athletes		Senior athletes	
	*k*	*N*	*k*	*N*
Age to reach performance milestones				
Overall	13	1728	23	1283
World class vs. national class			16	993
Lower-level comparisons			6	233
Main-sport starting age				
Overall	17	2336	40	2622
World class vs. national class			17	1030
Lower-level comparisons			22	1553
Main-sport coach-led practice				
Overall	39	4833	51	2997
World class vs. national class			17	1181
Lower-level comparisons			33	1759
Overall early	26	3134	37	2008
Main-sport peer-led play				
Overall	17	1680	23	1012
World class vs. national class			6	370
Lower-level comparisons			16	585
Overall early	13	1469	18	761
Other-sports practice and/or play				
Overall	18	2702	37	1957
World class vs. national class			18	1239
Lower-level comparisons			18	661
Overall early	15	1970	26	1513
Other-sports coach-led practice				
Overall	11	2324	22	1232
World class vs. national class			17	1002
Lower-level comparisons			5	218
Overall early	8	1592	20	1142
Other-sports peer-led play				
Overall	8	1592	18	1098
World class vs. national class			15	928
Lower-level comparisons			–	–
Overall early	–	–	8	508

*Early* activity only until age 15 years, *k* effect sizes, *N* sample size, *–* indicates not enough effect sizes (*k* < 5)

### Meta-Analytic Procedure

Data analyses included six successive steps:

1. We obtained the standardized mean difference between higher- and lower-performing athletes in a sample on one of our predictor variables and the corresponding sample size.
2. We tested whether extreme contrast comparisons yielded extreme effect sizes. We compared effect sizes between narrow, medium, and extreme performance bandwidths for main-sport practice, the predictor variable with sufficient effect sizes within each subsample (*k* ≥ 5), i.e., for each performance bandwidth among juniors and seniors. As expected, extreme contrasts produced extreme effect sizes (narrow: $\bar{d }$ = 0.29; 95% confidence interval [CI] 0.17–0.42, *p* < 0.001; medium: $\bar{d }$ = 1.08; 95% CI 0.53–1.63, *p* < 0.001; extreme contrast: $\bar{d }$ = 1.47; 95% CI 1.00–1.93, *p* < 0.001; *F* = 33.50, *p*  < 0.001). We thus excluded extreme contrast comparisons (*k* = 90) from the subsequent steps.
3. We searched for outliers, defined as Cohen’s *d* whose residuals had *z*-scores > 3 within junior and senior samples. Outliers (*k* = 10) were excluded from subsequent steps.
4. For each predictor variable, we estimated the overall effect (i.e., across age and performance levels) on performance differences and heterogeneity by conducting random-effects meta-analyses and then investigated whether some of the heterogeneity was explained by moderator variables using mixed-effect meta-analyses. We investigated moderator effects of age category (junior vs. senior athletes), types of sport, and—for senior athletes—absolute performance levels compared in each study (world class vs. national class and lower-level comparisons; preliminary analyses indicated that among junior samples there was no moderation effect of compared absolute performance levels, 0.019 < *F* < 3.329, all *p* > 0.05). For all moderator analyses, we used the rule of thumb that *k* ≥ 5 is required for each subgroup [36].
5. We investigated whether effects of different predictor variables were independent or associated with one another. We did this by computing the sample-weighted correlation coefficients (Pearson) among studies’ effect sizes (Cohen’s *d*) for the most relevant predictor variables. For example, are the effects of age to reach milestones and of starting age on eventual performance associated with one another?
6. We tested for potential publication bias.

We assessed the quality of evidence using the GRADE (Grading of Recommendations Assessment, Development, and Evaluation) system [41, 42]. This system is used for rating the quality of evidence in reviews and meta-analyses. The ratings provide systematic judgements on the confidence we can have that a true effect lies close to an effect estimate and classify the quality of a body of evidence into one of four levels: high, moderate, low, or very low. GRADE assesses study design, control of potential confounders, magnitude of effects, dose–response gradients, consistency of effects, directness and precision of the evidence, and publication bias. We rated the quality of evidence for each predictor variable, separately for junior performance, senior performance, and senior world-class versus national-class performance, and for the comparisons of predictor effects on junior versus senior performance.

All meta-analytic models were computed in the publicly available R environment using the “mixmeta 1.1.0” package [43, 44]. To compare coefficients in mixed-effect models, we applied Wald’s *F* test (package “car 3.0-10”) [45]. All hypothesis testing was two-tailed. A *p* value < 0.05 was considered statistically significant.

## Results

**Fig. 2:**
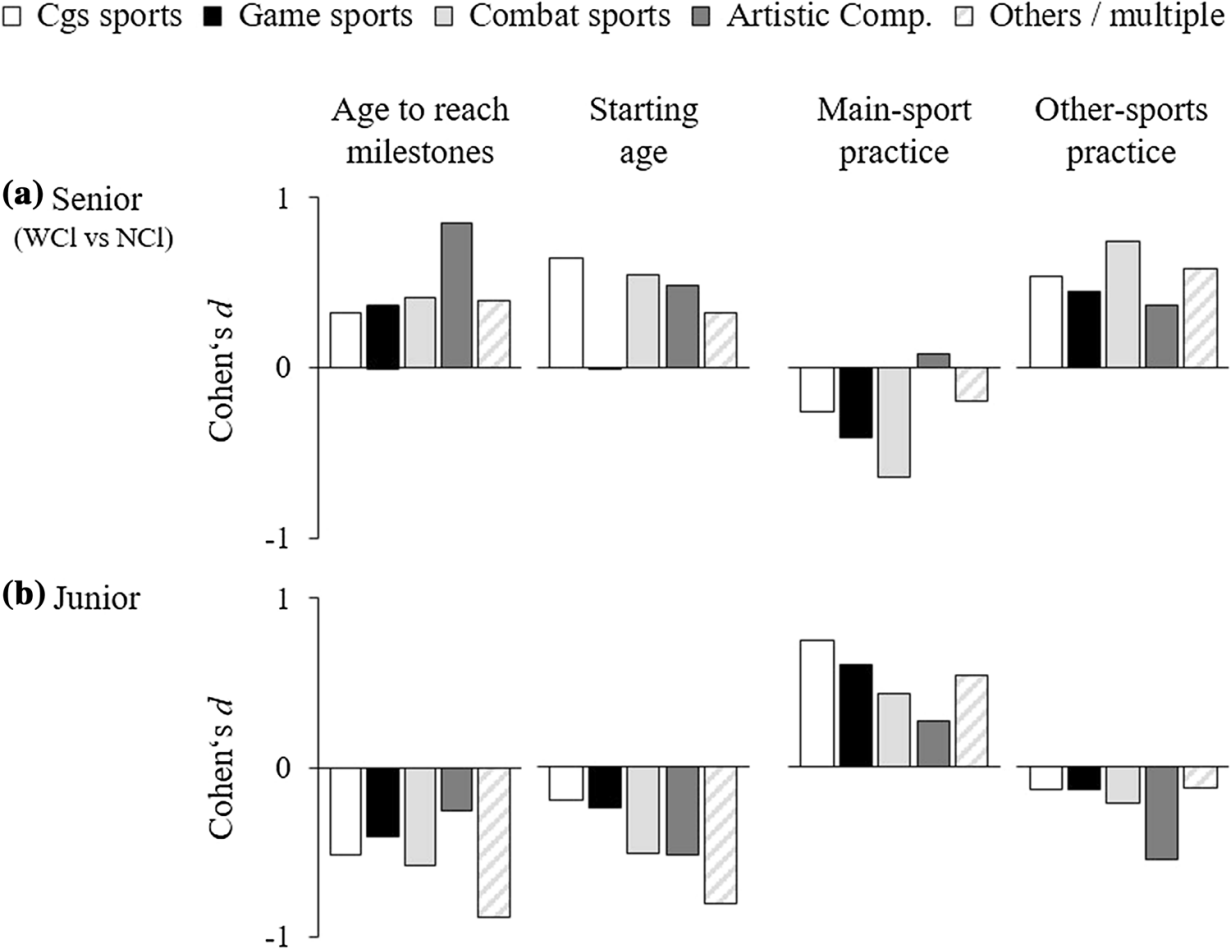
Effects of the most relevant predictors on performance in different types of sports. Comparisons between **a** senior WCl and NCl and **b** relatively higher- and lower-performing junior athletes. Cgs sports (white bars, *n* = 1420), game sports (black bars, *n* = 4106), combat sports (light grey bars, *n* = 174), artistic composition sports (dark grey bars, *n* = 141), and others/multiple sports (striped bars, *n* = 818). Note the sign of effects for age-related and activity-related predictors: A positive effect indicates that higher performance was associated with later (higher) ages and with greater activity amounts (and vice versa). 95% confidence interval omitted for clarity. *Cgs* performance is measured in centimeters, grams, or seconds, *NCl* national class, *WCl* world class

**Fig. 3:**
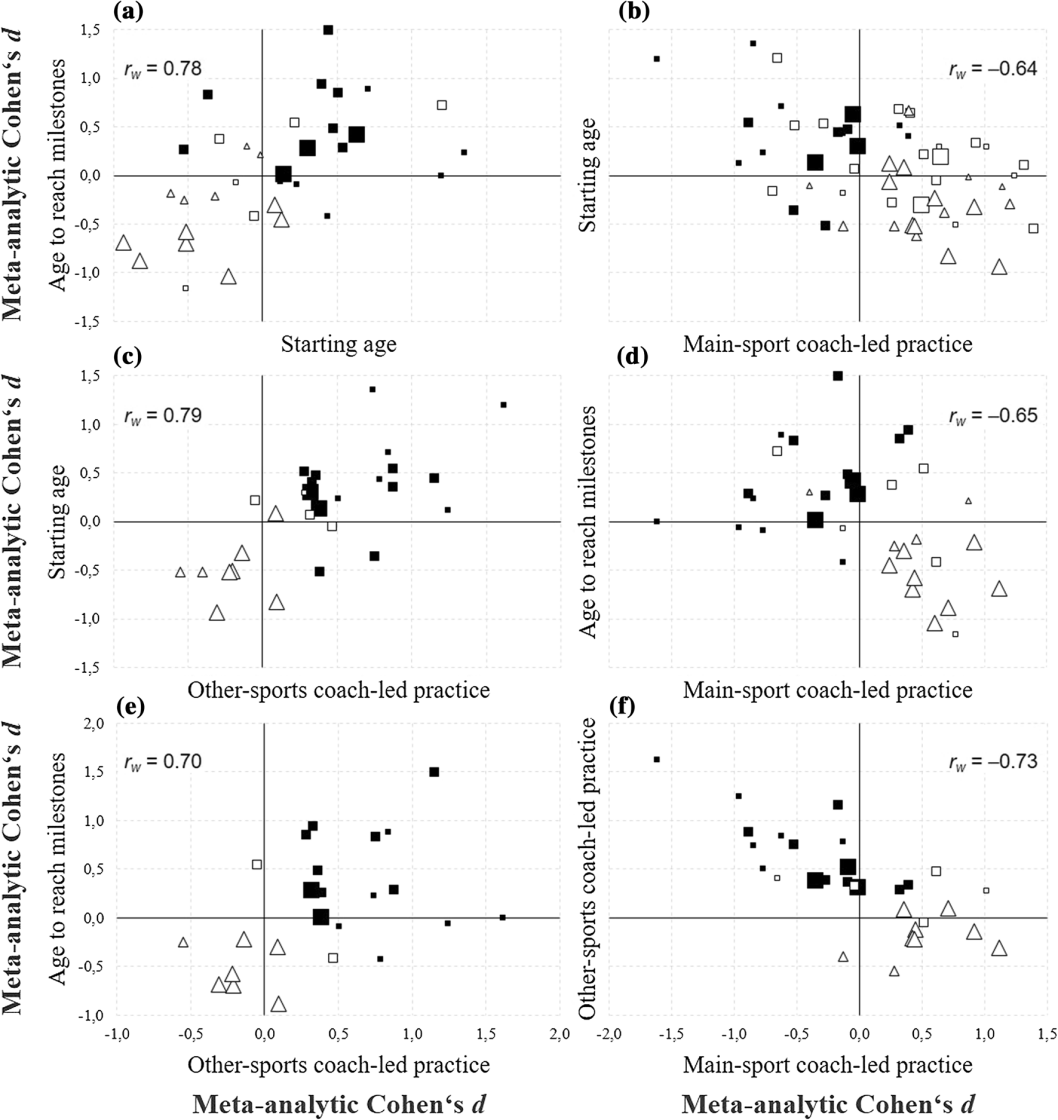
Associations between the effect sizes for the most relevant predictor variables. Each symbol represents a study. Note the sign of effects for age-related and activity-related predictors: a positive effect indicates that higher performance was associated with later (higher) ages and with greater activity amounts (and vice versa). Note that the correlations are between effect sizes of different predictor variables, not between individual athletes’ performance and their participation variables or between the predictor variables themselves. For example, the symbols in the top right quadrant of panel **a** represent studies where higher performance was associated with later age to reach milestones and later starting age. The symbols in the bottom left quadrant represent studies where higher performance was associated with earlier age to reach milestones and earlier starting age. The symbols in the top left quadrant of panel **f** represent studies where higher performance was associated with greater amounts of other-sports practice and smaller amounts of main-sport practice. The symbols in the bottom right quadrant represent studies where higher performance was associated with smaller amounts of other-sports practice and greater amounts of main-sport practice. *r*_w_ is the sample-weighted *r*, filled square senior world class vs. national class, unfilled square senior lower-level comparisons, unfilled triangle juniors. Large symbols indicate *n* > 100, medium symbols indicate 30 ≤ *n* ≤ 100, and small symbols indicate *n* < 30

The report of the results is structured following our three research questions. Regarding our first question, we report the overall findings for the entire sample (Table 4). Concerning our second question, we describe the results of the moderator analyses: we report whether effects differed between junior and senior samples (Table 4). Then, we document effects comparing senior world class and national class and effects involving lower-level comparisons (Table 5). Subsequently, we report the findings from different types of sports (Fig. 2). With respect to our third question, we describe the extent to which effects of different predictor variables are independent or associated with one another (Fig. 3). In addition, we assess the quality of evidence. Forest plots, *I*^2^ statistics, and publication bias analyses are reported in the ESM.

**Table 4:** Predictor effects on performance^a^

Predictor variables	Overall			Juniors			Seniors			Comparison	
	$\bar{d }$	95% CI	*p* value	$\bar{d }$	95% CI	*p* value	$\bar{d }$	95% CI	*p* value	*F*	*p* value
Age											
Milestones	0.025	− 0.18 to 0.23	0.817	− 0.493	− 0.66 to − 0.32	< 0.001	0.362	0.15 to 0.58	< 0.001	34.13	< 0.001
Start	0.073	− 0.06 to 0.21	0.278	− 0.330	− 0.51 to − 0.15	< 0.001	0.278	0.15 to 0.41	< 0.001	29.77	< 0.001
Main sport											
Practice	0.396	0.25 to 0.54	< 0.001	0.610	0.40 to 0.82	< 0.001	0.200	0.03 to 0.38	0.025	8.61	0.004
Early	0.194	0.05 to 0.34	0.009	0.533	0.36 to 0.71	< 0.001	− 0.099	− 0.26 to 0.06	0.237	26.44	< 0.001
Play	0.201	0.01 to 0.40	0.043	0.238	− 0.00 to 0.48	0.054	0.167	− 0.13 to 0.47	0.277	0.25	0.618
Early	0.170	− 0.02 to 0.36	0.081	0.180	− 0.01 to 0.37	0.068	0.142	− 0.19 to 0.47	0.398	0.21	0.649
Other sports											
Practice/play	0.078	− 0.03 to 0.18	0.137	− 0.159	− 0.32 to 0.00	0.056	0.235	0.14 to 0.33	< 0.001	28.87	< 0.001
Early	0.150	0.02 to 0.28	0.027	− 0.107	− 0.32 to 0.11	0.331	0.340	0.23 to 0.45	< 0.001	18.30	< 0.001
Practice	0.189	0.03 to 0.35	0.018	− 0.232	− 0.35 to − 0.12	< 0.001	0.465	0.35 to 0.58	< 0.001	68.53	< 0.001
Early	0.277	0.11 to 0.45	0.001	− 0.136	− 0.28 to 0.01	0.065	0.505	0.36 to 0.65	< 0.001	37.52	< 0.001
Play	0.001	− 0.09 to 0.09	0.992	− 0.121	− 0.22 to − 0.02	0.021	0.130	0.01 to 0.25	0.040	9.38	0.006
Early	0.105	− 0.03 to 0.24	0.114	–	–	–	0.153	− 0.03 to 0.33	0.095	–	–

Overall (junior and senior athletes) and among junior and senior athletes. Main-sport and other-sports practice and play: amounts accumulated through one’s entire career. “early” activities indicate the amount until age 15 years

*CI* confidence interval, $\bar{d }$ meta-analytic mean Cohen’s $\bar{d }$, – indicates not enough effect sizes (*k* < 5)

^a^Note the sign of effects for age- and activity-related predictors: a positive effect indicates that higher performance was associated with later (higher) ages and with greater activity amounts

**Table 5:** Predictor effects on senior performance among higher and lower absolute performance levels^a^

Predictor variables	World class vs. national class			Lower-level comparisons			Comparison	
	$\bar{d }$	95% CI	*p* value	$\bar{d }$	95% CI	*p* value	*F*	*p* value
Age								
Milestones	0.420	0.22 to 0.62	< 0.001	0.035	− 0.52 to 0.59	0.902	2.17	0.157
Start	0.409	0.19 to 0.63	< 0.001	0.174	− 0.00 to 0.35	0.055	2.67	0.111
Main sport								
Practice	− 0.232	− 0.38 to − 0.08	0.002	0.474	0.27 to 0.68	< 0.001	24.00	< 0.001
Play	− 0.033	− 0.30 to 0.23	0.811	0.143	− 0.25 to 0.54	0.478	0.17	0.682
Other sports								
Practice/play	0.269	0.15 to 0.39	< 0.001	0.140	− 0.02 to 0.30	0.076	1.67	0.205
Practice	0.497	0.37 to 0.63	< 0.001	0.208	− 0.06 to 0.48	0.130	3.58	0.074
Play	0.114	− 0.02 to 0.25	0.098	–	–	–	–	–

*CI* confidence interval, $\bar{d }$** =** meta-analytic mean Cohen’s $\bar{d },$ *–* indicates not enough effect sizes (*k* < 5)

^a^Note the sign of effects for age- and activity-related predictors: a positive effect indicates that higher performance was associated with later (higher) ages and with greater activity amounts

### Overall Effects and Effects Within Junior and Senior Samples

Table 4 shows the overall effects of the predictor variables (across junior and senior athletes and all performance categories), then within junior athletes and within senior athletes and comparisons between junior and senior athletes’ effects.

Earlier milestone achievement, earlier starting age, more main-sport practice, and less other-sports practice was associated with higher junior performance. In contrast, later milestone achievement, later starting age, and more other-sports practice was associated with higher senior performance. Furthermore, amount of main-sport practice was less predictive of senior performance than of junior performance; amount of early main-sport practice was not predictive at all of eventual senior performance. Peer-led play, whether main sport or other sports, had negligible effects on both junior and senior performance ($\bar{d }$ <|0.20| and/or *p* > 0.05).

### Effects Across Different Senior Performance Levels

Effects of the predictor variables on performance differences of senior world-class versus national-class athletes and on performance differences among lower-level comparisons (local up to national level) are displayed in Table 5. Senior world-class athletes significantly differed from their national-class counterparts in that world-class performers reached performance milestones later, started their main sport later, and accumulated significantly less main-sport practice but significantly more other-sports practice. Neither peer-led play in the main sport nor in other sports predicted differences between senior world-class and national-class athletes.

Effects generally did not differ significantly between studies comparing world-class and national-class athletes versus studies involving lower-level comparisons (Table 5), with one exception: main-sport coach-led practice. World-class performers accumulated significantly less main-sport practice than national-class athletes, whereas among lower-level comparisons, relatively higher-performing athletes accumulated more main-sport practice than their lower-performing counterparts.

### Types of Sports

We grouped types of sports based on the analytical categorization of the task in competition [25] (cgs, game, combat, artistic composition sports, and others). Figure 2 highlights the patterns of effects of the most relevant predictors within each type of sports, separately for samples involving junior athletes and those involving senior world-class and national-class athletes. At a descriptive level, the patterns of effects were generally similar across types of sports. Importantly, none of the types of sports displayed a contrary pattern of effects relative to the other types of sports.

Whenever enough effect sizes were available for a predictor variable within types of sports (*k* ≥ 5), we tested differences between types of sports for significance among junior and senior samples. We generally found no significant differences of effects between types of sports, with one exception: the effect size of starting age among senior world-class and national-class athletes differed between cgs and game sports ($\bar{d }$ **=** 0.65, 95% CI 0.32–0.99 vs. $\bar{d }$ **=** 0.00, 95% CI − 0.43 to 0.43, *F* = 6.36, *p* = 0.028).

### Associations Between Effects of Different Predictor Variables

Effect sizes from the most relevant predictor variables—age to reach milestones, starting age, amount of main-sport practice, and amount of other-sports practice—were not independent but were correlated with one another: |0.64|< *r*_w_ <|0.79|. Figure 3 illustrates how studies comparing senior world-class and national-class athletes and studies comparing higher and lower-performing junior athletes are mostly located in opposite quadrants in each of the panels (a) to (f). That is, different sample types defined by different age and performance levels tended to produce predictable patterns of effect sizes across predictor variables.

### Quality of Evidence

Based on our GRADE rating, the quality of evidence was moderate for the effects of age to reach milestones, starting age, main-sport practice, and other-sports practice regarding junior performance, senior performance, and differences of effects on junior versus senior performance (moderate study design; dose–response gradient; high consistency of effects; high directness; high precision; no publication bias; see Table S2 in the ESM for details). The quality of evidence for these predictors was moderate to high among senior world-class versus national-class comparisons (consistent findings from studies controlling for potential confounds). However, the quality of evidence was generally low for effects of main-sport play and other-sports play. This was due to negligibly small and inconsistent effects, yielding no dose–response gradient.

## Discussion

**Fig. 4:**
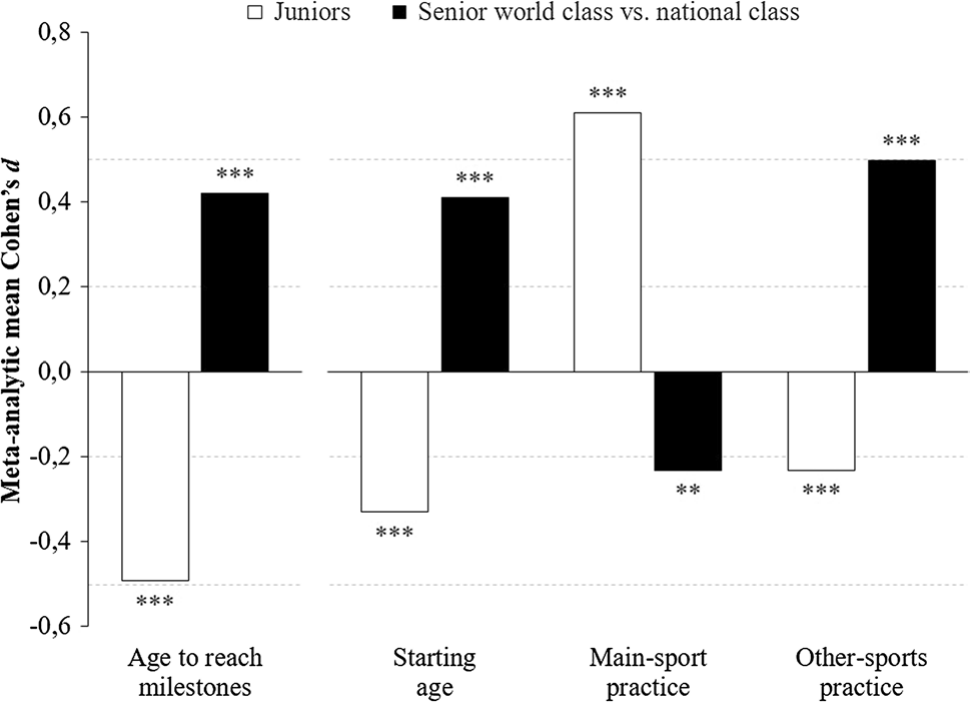
Overview of the central results. Effects of the most relevant predictor variables among senior world-class and national-class athletes (black bars) and among junior-age athletes (white bars). Note the sign of effects for age-related and activity-related predictors: A positive effect indicates that higher performance was associated with later (higher) ages and with greater activity amounts. **p < 0.01, ***p < 0.001; ~$\bar{d }$|0.20|= small effect, ~$\bar{d }$|0.50|= medium effect

The meta-analysis investigated the effects of childhood/adolescent progress and of amounts of practice and play activities, both in an athlete’s main sport and in other sports, on individual differences in eventual performance during junior and senior ages. Analyses considered the entire range of variables defining the multidimensional specialization–diversification continuum and involved a sample of 9241 athletes from a variety of sports, of both sexes, and competing at wide ranges of performance levels, from the local to the Olympic competition level. Figure 4 summarizes the major findings.

Across different types of sports, analyses revealed five central findings that answered our research questions.

1. Participation patterns predicted performance. Moreover, childhood/adolescent participation patterns predicted long-term adult performance.
2. Compared with their national-class counterparts, senior world-class athletes engaged in more childhood/adolescent coach-led practice in sports other than their main sport and, relatedly, began playing their main sport later; accumulated less main-sport practice; and reached performance milestones in their main sport at a slower rate.
3. Predictors of junior-age performance were opposite of those of senior-age performance: higher youth-age performance was associated with an earlier start of playing the main sport; greater amounts of main-sport practice but less other-sports practice; and a faster rate of achieving milestones.
4. Peer-led play in either the athlete’s main sport or in other sports had negligible effects on both junior and senior performance.
5. Effects of age to reach milestones, starting age, amount of main-sport practice, and amount of other-sports practice were not independent but were closely associated with one another.

The findings were robust across different types of sports and were also consistent with results from original studies that involved control for potential confounds through matched-pairs design, multi-year longitudinal quasi-experiments, and multivariate linear and non-linear analyses [24, 25, 27, 28, 46–48].

### Theoretical Implications

The reduced main-sport practice of senior world-class compared with national-class athletes does not call into question the effect of sport-specific practice on sport-specific performance and the importance of multi-year extensive main-sport coach-led practice. Indeed, all the senior world-class and national-class athletes had engaged in substantial main-sport practice. However, performance differences between athletes were parabolically, not monotonically, related to main-sport practice amounts: across athletes, increasing amounts of main-sport practice were associated with increasing performance from the local up to the national level; beyond national class, less main-sport practice was associated with higher performance.

Furthermore, both senior world-class and national-class performers had remarkable performance progression in their early years, but the early progress of national-class athletes was even faster. The data indicate that athletes whose development was particularly accelerated in childhood/adolescence—typically through intensive specialized main-sport practice—were frequent among the eventual senior national-class athletes (and also among the most successful junior athletes) but were infrequent among the eventual senior world-class performers.

How can this complex and partly counterintuitive pattern of findings be explained? In particular, why was senior world-class performance associated with less main-sport practice than national-class performance, why did childhood/adolescent non-specific practice facilitate later specific performance, and why did participation patterns have different short- and long-term effects? The aforementioned concepts—deliberate practice, deliberate play, and giftedness [8, 11, 16, 17]—are partly consistent with the predictors of junior performance. However, they do not provide adequate approaches to explain the highest adult performance levels, primarily because their central premises are inconsistent with the empirical evidence. Alternatively, we suggest that approaches from neoclassical economics may provide a fruitful heuristic, especially the concepts of efficiency and sustainability.

We illustrate with a practical example of a 14-year old athlete. Her absolute amount of disposable time is generally limited by the time demands of school, sleep, and meals. Within the available scope, if she expands the amount of specialized main-sport practice (e.g., from three to five weekly sessions), she will boost her current adaptations, learning, and short-term junior performance but may limit her scope for future long-term response to practice (adaptations, learning). At the same time, expanding the amount of specialized practice shifts her load–recovery balance towards the “load pole,” which increases her risk of overtraining and of later overuse injuries. In addition, the expanded time in main-sport practice reduces her available time for homework, study, playing other sports, other hobbies, and spending time with friends, family, or alone [25, 49, 50].

On the other hand, limiting herself to only three weekly main-sport sessions—and perhaps participating in multi-sport practice—may leave a greater scope for future long-term adaptations and learning, will reduce her risks of overtraining and (future) overuse injury, and give her more available time for education, friends, family, etc. However, she is foregoing the additional short-term adaptations and learning of expanded main-sport practice and is limiting her junior performance.

The example reflects only two patterns, but the implied question applies to the entire continuum of multi-dimensional participation patterns: what total amount and what amounts of the different types of sport activities at which ages lead to which performance improvement and at which age?

The neoclassical economics perspective implies several relevant premises:

1. The goal is to achieve success at international senior championships, which has a greater value than junior success (publicity, prestige, income of prize money, sponsorships, public funding). However, international senior success is an extremely scarce good that many compete for but only few achieve. An athlete’s career is therefore characterized by great uncertainty of success.
2. Resources are restricted and must be economized: available time (time demands of education, sleep, meals; length of athletic career), the athlete’s body, load tolerance, health, coaching, facilities.
3. Sport activities yield short- and long-term benefits, costs, and risks. For example, during childhood/adolescence, greater amounts of previous main-sport practice are typically associated with higher current performance. But the accumulated main-sport practice is also associated with the costs of diminishing future response to practice, accumulating opportunity costs (i.e., the lost benefit of foregone other activities), and increased (future) risks of overtraining and overuse injury. Coaches and athletes pursue the participation pattern that yields the optimal ratio of benefits, costs, and risks over the short and long term; i.e., a classical problem of the optimization of the allocation of resources. Further, benefits, costs, and risks differ over the short and long term; i.e., a classical problem of sustainability.
4. Because (1) resources are limited and (2) one endeavors to increase benefits while limiting costs and risks, the efficiency of practice is paramount. In economic terms: the marginal productivity, Δ performance / Δ practice over time, see Eq. 1. Following the Gossenian law of diminishing marginal productivity [51, 52], the more main-sport practice previously accumulated, the lower the added gain in performance per added unit of main-sport practice (see Eq. 2).
5. The higher the competition level, the greater the value of every unit of absolute performance improvement: small differences in absolute performance (velocity run, distance jumped, successful shots made) make great differences to an athlete’s championship level and placing, i.e., relative performance. For example, at an international level, 0.1 s in a race may distinguish the gold medalist from a non-medalist. In economic terms, the marginal revenue product increases with age and competition level (see Eq. 3).
  1 $$\begin{aligned} &{\text{Marginal productivity}} \\ & \;\; = \frac{{{\text{performance }}\,t_{{\text{i}}} - {\text{performance}}\, t_{{{\text{i}} - 1}} }}{{{\text{practice amount}}\, t_{{\text{i}}} - {\text{practice amount}}\, t_{{{\text{i}} - 1}} }} \\ & \;\; = \frac{{\Delta {\text{performance}}}}{{\Delta {\text{practice amount}}}} \\ \end{aligned}$$
  2 $$\begin{aligned} & {\text{Diminishing marginal productivity:}} \\ & \;\; \frac{{\Delta\, {\text{performance }}\,t_{{\text{i}}} }}{{\Delta {\text{practice amount}} \,{{t}}_{{\text{i}}} }} < \frac{{\Delta\, {\text{performance}}\, t_{{{\text{i}} - 1}} }}{{\Delta {\text{practice amount}} \,{{t}}_{{{\text{i}} - 1}} }} \end{aligned}$$
  3 $$\begin{aligned} & {\text{Marginal revenue product }} \\ & \;\; = {\text{ marginal productivity}}\times {\text{value}} \\ & \;\; = \frac{{\Delta\, {\text{ absolute performance }} \times {\text{value}}}}{{\Delta\, {\text{ practice amount}}}} \\ & \;\; = \frac{{\Delta \,{\text{relative }} {\text{performance}}}}{{\Delta {\text{practice amount}}}} \\ & \;\; = \frac{{\Delta {\text{competition level }}\left( {{\text{championship level}};{\text{ placing}}} \right)}}{{\Delta\, {\text{practice amount}}}} \\ \end{aligned}$$

**Fig. 5:**
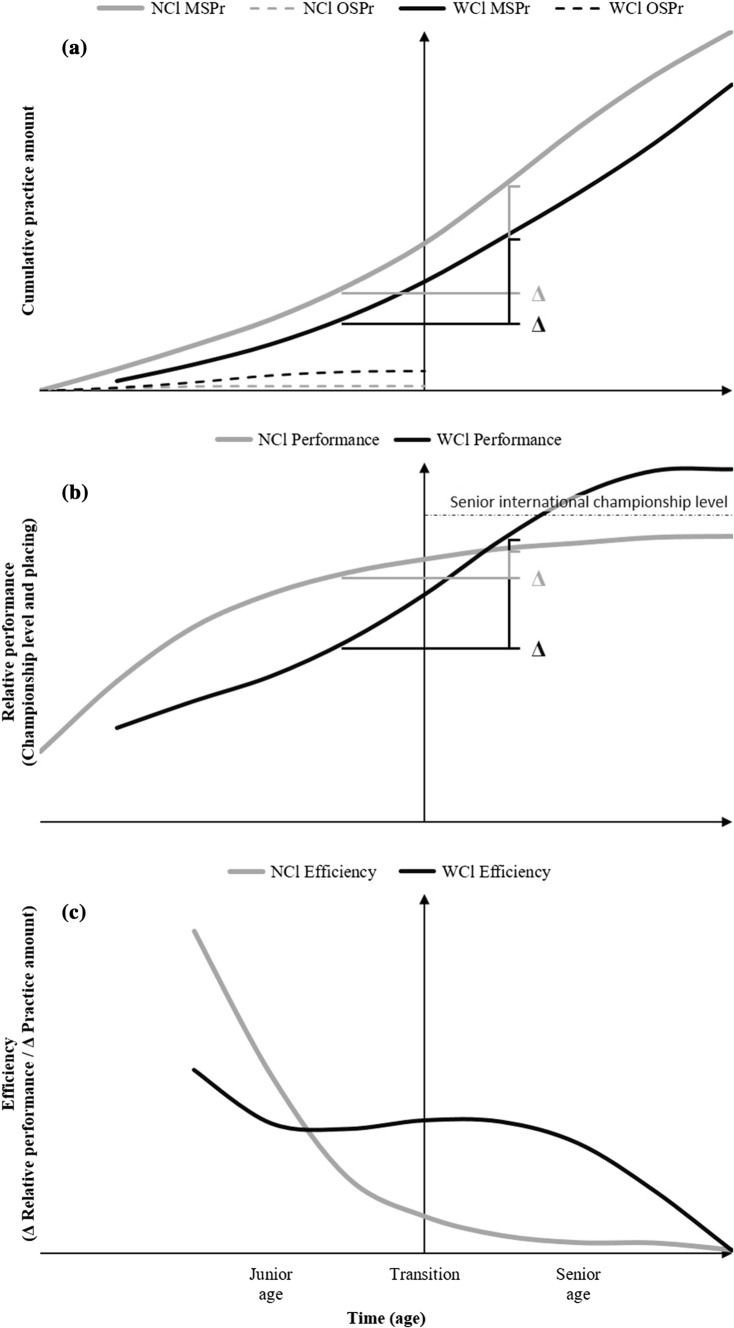
Practice amounts, performance and efficiency of practice across age of the senior WCl and NCl athletes—schematic illustration. The y-axis (center) marks the junior age limit (18–19 years in most sports). **a** Amounts of MSPr and OSPr. The *triangles* illustrate the *Δ* practice amount per time unit. **b** Performance; the triangles illustrate the *Δ* performance per time unit. Note that relative performance was measured as championship level and placing, not athletes’ absolute velocity run, distance jumped, etc. The championship level and placing reflects the differences between an athlete’s and opponents’ absolute performance. **c** Efficiency of practice (marginal productivity × value), i.e., Δ relative performance/Δ practice amount per time unit. Note that WCl athletes’ increasing efficiency of practice in adulthood includes the increasing marginal revenue product, i.e., the fact that, in adult high-performance sports, small differences in improvement of absolute performance lead to substantial differences in improvement of championship level and placing. *NCl* national class, *WCl* world class, *MSPr *main-sport practice, *OSPr* other-sports practice

To reflect our empirical findings in view of these economic premises, Fig. 5 displays a schematic illustration based on data from primary studies. The figure shows the senior world-class and national-class athletes’ amounts of main-sport and other-sports coach-led practice, development of relative performance (competition level and placing), and their efficiency of practice over time.

Compared with their national-class counterparts, world-class athletes generally invested less time in main-sport coach-led practice throughout their career but more time in childhood/adolescent other-sports coach-led practice (Fig. 5a). The world-class performers initially had a slower performance progress but greater improvement of relative performance during late adolescence and adulthood than national-class athletes (Fig. 5b). That is, the world-class athletes’ combination of reduced childhood/adolescent investment in specialized main-sport practice with greater diversified engagement in multi-sport practice was associated with lower initial marginal productivity, but greater sustainability, in that it yielded greater long-term marginal productivity (efficiency of practice, Δ relative performance/Δ practice amount, Fig. 5c). Whereas the national-class athletes exhibited a continuous decline of marginal productivity over time, the world-class athletes increased their marginal productivity of relative performance, i.e., marginal productivity × value—championship level and placing.

Notably, world-class athletes’ enhanced efficiency of practice was exactly located in the age period and competition level of the greatest marginal revenue product, i.e., where relatively small differences in absolute performance made great differences in championship level and placing (relative performance).

The findings also indicate that, whereas senior world-class athletes did not have greater initial potential (i.e., no faster initial progress) than their national-class counterparts, they differed in two other respects: first, eventual national-class athletes depleted a large part of their initial potential during junior age, whereas world-class athletes depleted a smaller part of their initial potential during junior age. Second, unlike the national-class athletes, the world-class athletes simultaneously expanded their potential to respond to subsequent long-term practice (i.e., response in terms of physical and physiological adaptations, perceptual–motor learning).

The economic description raises the question of why and how childhood/adolescent multi-sport practice expands athletes’ potential and facilitates their long-term efficiency of main-sport practice and sustainable performance development. Our economic interpretation is underpinned by three inter-related hypotheses discussed in the literature:

1. Childhood/adolescent multi-sport engagement is associated with reduced risks of later overuse injuries and burnout (see Bell et al. [50] and Waldron [53] for reviews).
2. Practice and competition experiences in various sports increase the odds that athletes find the sport that best matches their talent and preferences (economic “search and match theory” [54, 55]; in sports [28, 48]). Individual preferences may include enjoyment, coach–athlete relationship, and peer interaction in a sport, among others. A priori, an athlete’s information on athlete–sports match is vague but is expanded through gaining experience in various sports. That is, the “talent identification” for a sport occurs a posteriori not a priori [56, 57].
  According to this hypothesis, the (few) senior world-class athletes who specialized early were either talented at multiple sports or fell into an individually suitable sports match largely by luck.
3. Varied learning experiences (tasks, situations, methodologies) may expand the potential for future long-term learning, i.e., one’s learning capital, in two related ways (i.e., the “learning transfer as preparation for future learning” hypothesis [58]). First, varied learning experiences facilitate the athlete’s ability to adapt to different learning tasks, situations, methodologies, and available information for learning. The athlete becomes a more adaptive learner and can better exploit learning opportunities [59, 60]. Second, the athlete experiences various learning designs that vary in efficacy for the individual athlete; these experiences help them understand individually more and less athlete-functional learning solutions [48, 58]. At the same time, these experiences may facilitate the athlete’s competencies for self-regulation in learning (see Jordet [61] for a review).

According to these hypotheses, athletes who engage in excess childhood/adolescent specialized main-sport practice may more likely be hampered by (later) overuse injuries and/or burnout, may have a greater risk of “malinvestment” in a suboptimal sports match, and may have limited opportunities to expand their learning capital for future long-term learning. In contrast, senior world-class athletes’ pattern of childhood/adolescent multi-sport practice with relatively less main-sport practice was likely associated with reduced risks of (later) overuse injury and/or burnout, increased odds that they selected a main sport at which they are particularly talented, and improved long-term perceptual–motor skill learning.

The hypotheses are also supported by three findings from several previous studies [22, 25, 26, 31, 46–48, 62–66]: (1) Childhood/adolescent multi-sport practice did not have a direct effect on main-sport performance but had a delayed moderator effect, such that it facilitated the athlete’s later main-sport efficiency of practice; (2) the effect rested on improved later perceptual–motor learning, not physical development; (3) the effect was not moderated by the relatedness of the different sports an athlete engaged in. Further, the present finding that multi-sport coach-led practice, but not peer-led play, facilitated long-term senior performance provides additional support for our second and third hypotheses.

Collectively, the findings suggest that a childhood/adolescent investment pattern that is relatively resource preserving (time, the athlete’s body, health), cost reducing (opportunity costs, future potential, especially learning capital), and risk buffering (injury, burnout, distributing “risk capital” to various sports) is associated with gradual initial progress but sustainable long-term efficiency of practice and performance development.

The arguments so far have concerned the micro-economic, individual athlete level. At a collective, macro-economic level, the findings suggest that a greater number of young athletes engaging in more childhood/adolescent multi-sport practice will expand the talent pool in a nation’s sport system.

### Methodological Considerations

The study has several strengths, such as a large international sample spanning wide ranges of sports, age categories, and performance levels, the analysis of the full range of predictor variables defining the specialization–diversification continuum, the examination of effects on performance at different age and performance levels and in different types of sports, and the investigation of associations between effects of various predictors. But it does have limitations. First, the retrospective and correlational design of many original studies may not have controlled for potential confounds or selection effects (e.g., survivor bias). Thus, although temporal precedence of activities on later performance is suggestive, the original study designs do not allow us to draw causal conclusions. Nevertheless, the major findings are entirely consistent with recent studies that controlled for potential confounds through matched-pairs designs and multivariate analyses, including multi-year quasi-experiments [24–28, 46–48, 64]. Second, we did not consider the “micro-structure” of an athlete’s main-sport practice (e.g., types of exercises, ways of executing them). However, several studies reported consistent findings from athletes who participated in the same training groups and thus the same main-sport practice [22, 26, 31, 67–70]. Third, we did not analyze potential interactions of other factors with participation patterns, such as athletes’ genotype, gene–environment interaction, familial support, or psychological characteristics. Fourth, male samples were over-represented and female samples were under-represented (67 and 33% of all participants, respectively). Other than age, sex, and country, studies generally did not report further demographic characteristics relevant to diversity. Additionally, relatively few studies involved samples from Africa and Central/South America, from combat sports and artistic composition sports, and from Paralympic sports. Furthermore, among junior game-sport samples, male soccer players were over-represented. Fifth, sample sizes and statistical power varied across meta-analytic models. Sixth, as in any systematic review and meta-analysis, although we used multiple databases, bias of availability, country, and language was possible. Finally, the quality of evidence was low for main-sport play and other-sports play.

### Future Directions

Researchers should seek to extend investigations to populations that are under-represented in present research, especially female athlete samples, samples from Africa and Central/South America, samples from Paralympic sports, and samples from combat and artistic composition sports. Among juniors, research into populations other than male youth soccer players should be expanded.

Our findings emphasize that predictors of the highest senior performance levels cannot be extrapolated from findings from lower performance levels or junior samples. Thus, the goal for future research is to further investigate participation patterns leading to the highest senior performance levels, rather than only junior-level performance. This includes further investigation into the causal processes underlying differing short- and long-term effects of different participation patterns.

We suggest that economic principles, particularly the concepts of efficiency and sustainability, provide a fruitful heuristic. This heuristic stipulates the investigation of the following questions for different participation patterns: (1) Which short- and long-term benefits, costs, and risks does a participation pattern yield; (2) to what magnitude and to what probability does a participation pattern yield those benefits, costs, and risks; (3) what (material and immaterial) value does each of these benefits, costs, and risks have; and (4) what is the eventual ratio of the summed value of all benefits relative to the summed value of all costs and risks accumulated through an athletic career?

Additionally, the associations between effects of different predictor variables call for the consideration of interactions between participation variables (and maybe with others, including social–environmental, psychological, or genetic factors), perhaps combining traditional statistics with advanced methods allowing multivariate non-linear analyses (for recent examples, see Barth et al. [27] and Barth and Güllich [28]). Such research may advance a theory of practice and of the development of expertise that considers efficiency and sustainability, explains short- and long-term effects, and allows the researcher to estimate optimal allocations of resources at both the individual and the collective level.

## Supplementary Information

Below is the link to the electronic supplementary material.

Supplementary file1 (PDF 2756 KB)

## Declarations

### Availability of data and material

Additional supporting information and the original data are freely available at https://osf.io/5bexm/?view_only=ccf48c64b6a0456e8022aee08715356a.
