# Correction to: Predictors of Junior Versus Senior Elite Performance are Opposite: A Systematic Review and Meta-Analysis of Participation Patterns

**DOI:** 10.1007/s40279-022-01653-8

**Published:** 2022-02-07

**Authors:** Michael Barth, Arne Güllich, Brooke N. Macnamara, David Z. Hambrick

**Affiliations:** 1 Department of Business and Society, University of Applied Sciences Kufstein Tyrol, Kufstein, Austria; 2 Department of Sport Science, University of Innsbruck, Innsbruck, Tyrol Austria; 3 Department of Sports Science, University of Technology Kaiserslautern, Kaiserslautern, Germany; 4 Department of Psychological Sciences, Case Western Reserve University, Cleveland, OH USA; 5 Department of Psychology, Michigan State University, East Lansing, MI USA

## Correction to: Sports Medicine 10.1007/s40279-021-01625-4

This article was initially published online on 17 January 2022. Due to a problem that occurred during production, the published article included a number of minor typographical errors. The published version of this article has since been updated to have these errors corrected. Below is a list of the errors that were corrected. Page numbers refer to the pdf version of the article.

- Page 3, first column, line 11: “effects sizes” was changed to “effect sizes”.
- Page 6, second column, line 11: “(… [CI] 0.17–0.42), … *p* < 0.001).” A parenthesis was removed as to read: “(…[CI] 0.17–0.42, … *p* < 0.001).”
- Page 8, Table 5, row headings: “Main-sport” was replaced with “Main sport”; “Other-sports” was replaced with “Other sports”.
- Page 9, second column, line 11: “(… –0.43 to 0.43), … *p* = 0.028).” A parenthesis was removed as to read: “(… –0.43 to 0.43, … *p* = 0.028).”
- Page 11, first column, line 15: “age” was replaced with “ages”.
- Page 13, second column: the horizontal line above Eq. (2) was removed.
- Page 13, Eq. (3): “marginal productivity value” was replaced with “marginal productivity × value”.
- Page 13, Eq. (3): The alignment of this equation was corrected.

The original article has been updated.

**Open Access** This article is licensed under a Creative Commons Attribution 4.0 International License, which permits use, sharing, adaptation, distribution and reproduction in any medium or format, as long as you give appropriate credit to the original author(s) and the source, provide a link to the Creative Commons licence, and indicate if changes were made. The images or other third party material in this article are included in the article's Creative Commons licence, unless indicated otherwise in a credit line to the material. If material is not included in the article's Creative Commons licence and your intended use is not permitted by statutory regulation or exceeds the permitted use, you will need to obtain permission directly from the copyright holder. To view a copy of this licence, visit http://creativecommons.org/licenses/by/4.0/.

