# Supplementary material for: Predictors of Junior Versus Senior Elite Performance are Opposite: A Systematic Review and Meta-Analysis of Participation Patterns
Source: Sports Med. 2022 Jan 17;52(6):1399–416. doi: 10.1007/s40279-021-01625-4 (PMC9124658; doi:10.1007/s40279-021-01625-4)
Supplement: Supplementary file 1 — Supplementary file1 (PDF 2756 KB) [file 40279_2021_1625_MOESM1_ESM.pdf]

Predictors of junior versus senior elite performance are opposite: A systematic review and meta-analysis of participation patterns – Supplementary information

Sports Medicine

Michael Barth[(a)]*, Arne Güllich[(b)], Brooke N. Macnamara[(c)], David Z. Hambrick[(d)]

[(a)] Department of Business & Society
University of Applied Sciences Kufstein Tirol
Andreas Hofer-Straße 7
6330 Kufstein
Austria

ORCID ID http://orcid.org/0000-0001-5333-3122

* correspondence to Michael Barth: michael.barth@fh-kufstein.ac.at

[(b)] Department of Sports Science
University of Technology Kaiserslautern
Erwin-Schrödinger-Straße 57
67663 Kaiserslautern
Germany

ORCID ID http://orcid.org/0000-0001-6911-2236

[(c)] Department of Psychological Sciences
Case Western Reserve University
11220 Bellflower Road
Cleveland, OH, 44106
USA

ORCID ID https://orcid.org/0000-0003-1056-4996

[(d)] Department of Psychology
Michigan State University
316 Physics Road
East Lansing, MI, 48825
USA

ORCID ID https://orcid.org/0000-0002-5315-5597

Tab. S1. Description and results of the primary studies.

Ref. N°	Reference	Study N°	Age_Cat	Sex	Country	Sports [a]	Method definition performance	Method data collection	Method data analysis	Published
1	Baker et al., 2003a	1	Senior	mf	AUS	Game sports: BB, FH, NB	coach evaluation	interview	group comp	publ
2	Baker et al., 2003b	2	Senior	mf	AUS	Game sports: BB, FH, NB	coach evaluation	interview	group comp	publ
3	Baker et al., 2005_s1	3	Senior	m	CAN	Triathlon	competition level	interview	group comp	publ
3	Baker et al., 2005_s2	4	Senior	m	CAN	Triathlon	competition level	interview	group comp	publ
3	Baker et al., 2005_s3	5	Senior	m	CAN	Triathlon	competition level	interview	group comp	publ
4	Baker et al., 2006_s1	6	Senior	m	CAN	Triathlon	competition level	interview	group comp	publ
4	Baker et al., 2006_s2	7	Senior	m	CAN	Triathlon	competition level	interview	group comp	publ
4	Baker et al., 2006_s3	8	Senior	m	CAN	Triathlon	competition level	interview	group comp	publ
5	Baker et al., 2012_s1	9	Junior	m	GER	Handball	coach evaluation	interview	group comp	publ
5	Baker et al., 2012_s2	10	Junior	f	GER	Handball	coach evaluation	interview	group comp	publ
6	Barreiros et al., 2013	11	Senior	mf	POR	Multiple sports	competition level	interview	group comp	publ
7	Berry et al., 2008	12	Senior	m	AUS	AUS football	coach evaluation	interview	group comp	publ
8	Bjoerndal et al., 2016_s1	13	Junior	f	NOR	Handball	competition level	questionnaire	group comp	publ
8	Bjoerndal et al., 2016_s2	14	Junior	f	NOR	Handball	competition level	questionnaire	group comp	publ
8	Bjoerndal et al., 2016_s3	15	Junior	f	NOR	Handball	competition level	questionnaire	group comp	publ
9	Bruce et al., 2013	16	Senior	f	AUS	Netball	coach evaluation	questionnaire	group comp	publ
10	Casado et al., 2019_s1	17	Senior	m	ESP, KEN	Athletics (long distance)	competition level	questionnaire	group comp	publ
10	Casado et al., 2019_s2	18	Senior	m	ESP, KEN	Athletics (long distance)	competition level	questionnaire	group comp	publ
10	Casado et al., 2019_s3	19	Senior	m	ESP	Athletics (long distance)	competition level	questionnaire	group comp	publ
11	Cathey & French, 2014	20	Senior	m	USA	Baseball (pitchers)	competition level	interview	group comp	publ
12	Cathey, 2010	21	Senior	m	USA	Baseball (pitchers)	competition level	interview	group comp	unp
13	Coelho-e-Silva et al., 2012	22	Junior	m	POR	Roller hockey	coach evaluation	questionnaire	group comp	publ
14	Coutinho et al., 2014	23	Senior	mf	POR	Volleyball	competition level	questionnaire	group comp	publ
15	Coutinho et al., 2015_s1	24	Senior	m	POR	Volleyball	competition level	interview	group comp	publ
15	Coutinho et al., 2015_s2	25	Senior	f	POR	Volleyball	competition level	interview	group comp	publ
16	Coutinho et al., 2016_s1	26	Senior	m	POR	Volleyball	competition level	interview	group comp	publ
16	Coutinho et al., 2016_s2	27	Senior	f	POR	Volleyball	competition level	interview	group comp	publ
17	Da Matta, 2004	28	Senior	f	BRA	Volleyball	coach evaluation	interview	group comp	unp
18	Dehghansai et al., 2016	29	Junior	mf	CAN	Wheelchair basketball	competition level	questionnaire	group comp	unp
19	Drake & Breslin, 2017	30	Senior	f	IRE	Field hockey	coach evaluation	questionnaire	group comp	publ
20	Duffy et al., 2004_s1	31	Senior	m	GBR	Dart	competition level	interview	group comp	publ
20	Duffy et al., 2004_s2	32	Senior	f	GBR	Dart	competition level	interview	group comp	publ
21	Elferink-Gemser et al., 2004_s1	33	Junior	f	NED	Field hockey	coach evaluation	questionnaire	group comp	publ
21	Elferink-Gemser et al., 2004_s2	34	Junior	m	NED	Field hockey	coach evaluation	questionnaire	group comp	publ
22	Elferink-Gemser et al., 2006_s1	35	Junior	f	NED	Field hockey	coach evaluation	questionnaire	group comp	publ
22	Elferink-Gemser et al., 2006_s2	36	Junior	m	NED	Field hockey	coach evaluation	questionnaire	group comp	publ
23	Elferink-Gemser et al., 2011_s1	37	Junior	mf	NED	Field hockey	coach evaluation	questionnaire	group comp	publ
23	Elferink-Gemser et al., 2011_s2	38	Junior	mf	NED	Field hockey	coach evaluation	questionnaire	group comp	publ

Ref. N°	Reference	Study N°	Age_Cat	Sex	Country	Sports [(a)]	Method definition performance	Method data collection	Method data analysis	Published
24	Erikstad et al., 2018	39	Junior	m	NOR	Soccer	coach evaluation	questionnaire	group comp	publ
25	Ford & Williams, 2012	40	Junior	m	GBR	Soccer	coach evaluation	questionnaire	group comp	publ
26	Ford et al., 2009_s1	41	Junior	m	GBR	Soccer	coach evaluation	questionnaire	group comp	publ
26	Ford et al., 2009_s2	42	Junior	m	GBR	Soccer	coach evaluation	questionnaire	group comp	publ
27	Ford et al., 2010	43	Junior	m	GBR	Cricket (batsmen)	lab testing	questionnaire	group comp	publ
28	Guimaraes et al., 2019	44	Junior	m	POR	Basketball	lab testing	n.a.	correl	publ
29	Güllich & Emrich, 2014_s1	45	Senior	mf	GER	Cgs sports	competition level	questionnaire	group comp	publ
29	Güllich & Emrich, 2014_s10	46	Junior	mf	GER	Other types sports	competition level	questionnaire	group comp	unp
29	Güllich & Emrich, 2014_s11	47	Junior	mf	GER	Cgs sports	competition level	questionnaire	group comp	unp
29	Güllich & Emrich, 2014_s12	48	Junior	mf	GER	Game sports	competition level	questionnaire	group comp	unp
29	Güllich & Emrich, 2014_s13	49	Junior	mf	GER	Combat sports	competition level	questionnaire	group comp	unp
29	Güllich & Emrich, 2014_s14	50	Junior	mf	GER	Art. comp. sports	competition level	questionnaire	group comp	unp
29	Güllich & Emrich, 2014_s15	51	Junior	mf	GER	Other types sports	competition level	questionnaire	group comp	unp
29	Güllich & Emrich, 2014_s2	52	Senior	mf	GER	Game sports	competition level	questionnaire	group comp	publ
29	Güllich & Emrich, 2014_s3	53	Senior	mf	GER	Combat sports	competition level	questionnaire	group comp	publ
29	Güllich & Emrich, 2014_s4	54	Senior	mf	GER	Art. comp. sports	competition level	questionnaire	group comp	publ
29	Güllich & Emrich, 2014_s6	55	Junior	mf	GER	Cgs sports	competition level	questionnaire	group comp	publ
29	Güllich & Emrich, 2014_s7	56	Junior	mf	GER	Game sports	competition level	questionnaire	group comp	publ
29	Güllich & Emrich, 2014_s8	57	Junior	mf	GER	Combat sports	competition level	questionnaire	group comp	publ
29	Güllich & Emrich, 2014_s9	58	Junior	mf	GER	Art. comp. sports	competition level	questionnaire	group comp	publ
30	Güllich et al., 2020	59	Junior	m	GER	Soccer	competition level	questionnaire	correl	unp
31	Güllich, 2014	60	Senior	m	GER	Field hockey	competition level	questionnaire	group comp	publ
32	Güllich, 2017_s1	61	Senior	mf	GER	Cgs sports	competition level	questionnaire	group comp	publ
32	Güllich, 2017_s2	62	Senior	mf	GER	Game sports	competition level	questionnaire	group comp	publ
32	Güllich, 2017_s3	63	Senior	mf	GER	Combat sports	competition level	questionnaire	group comp	publ
32	Güllich, 2017_s4	64	Senior	mf	GER	Art. comp. sports	competition level	questionnaire	group comp	publ
32	Güllich, 2017_s5	65	Senior	mf	GER	Other types sports	competition level	questionnaire	group comp	publ
33	Güllich, 2018a_s1	66	Senior	mf	GER	Athletics	competition level	questionnaire	group comp	publ
33	Güllich, 2018a_s2	67	Senior	mf	GER	Athletics	competition level	questionnaire	group comp	unp
33	Güllich, 2018a_s3	68	Junior	mf	GER	Athletics	competition level	questionnaire	group comp	publ
33	Güllich, 2018a_s4	69	Junior	mf	GER	Athletics	competition level	questionnaire	group comp	unp
34	Güllich, 2019	70	Senior	f	GER	Soccer	competition level	questionnaire	group comp	publ
35	Gunn et al., 2019	71	Senior	mf	USA	Swimming	competition level	questionnaire	correl	unp
36	Hardy et al., 2013	72	Senior	mf	GBR	Multiple sports	competition level	interview	group comp	unp
37	Harris, 2008	73	Senior	n.a.	USA	Bowling	competition level	questionnaire	group comp	unp
38	Haugaasen et al., 2014	74	Junior	m	NOR	Soccer	coach evaluation	questionnaire	group comp	publ
39	Helsen et al., 1998_s1	75	Senior	m	BEL	Soccer	competition level	questionnaire	group comp	publ
39	Helsen et al., 1998_s2	76	Senior	m	BEL	Soccer	competition level	questionnaire	group comp	publ

Ref. N°	Reference	Study N°	Age_Cat	Sex	Country	Sports [(a)]	Method definition performance	Method data collection	Method data analysis	Published
39	Helsen et al., 1998_s3	77	Senior	m	BEL	Soccer	competition level	questionnaire	group comp	publ
39	Helsen et al., 1998_s4	78	Senior	m	BEL	Field hockey	coach evaluation	questionnaire	group comp	publ
39	Helsen et al., 1998_s5	79	Senior	m	BEL	Field hockey	competition level	questionnaire	group comp	publ
39	Helsen et al., 1998_s6	80	Senior	m	BEL	Field hockey	coach evaluation	questionnaire	group comp	publ
40	Hendry & Hodges, 2018_s1	81	Senior	m	GBR	Soccer	coach evaluation	questionnaire	group comp	publ
40	Hendry & Hodges, 2018_s2	82	Junior	m	GBR	Soccer	coach evaluation	questionnaire	group comp	publ
41	Hendry et al., 2018_s1	83	Senior	m	GBR	Soccer	coach evaluation	questionnaire	correl	publ
41	Hendry et al., 2018_s2	84	Junior	m	GBR	Soccer	coach evaluation	questionnaire	correl	publ
42	Hendry et al., 2019a	85	Junior	m	CAN, GBR	Soccer	coach evaluation	questionnaire	group comp	publ
43	Hendry et al., 2019b	86	Senior	f	CAN	Soccer	competition level	questionnaire	group comp	publ
44	Hendry, 2012_s1	87	Junior	m	GBR	Soccer	coach evaluation	questionnaire	correl	unp
44	Hendry, 2012_s2	88	Junior	m	GBR	Soccer	coach evaluation	questionnaire	correl	unp
44	Hendry, 2012_s3	89	Junior	m	GBR	Soccer	coach evaluation	questionnaire	correl	unp
45	Hodges et al., 2015	90	Senior	mf	CAN	Triathlon	competition level	questionnaire	correl	publ
46	Hopwood et al., 2015_s1	91	Senior	mf	AUS, CAN	Multiple sports	competition level	questionnaire	group comp	publ
46	Hopwood et al., 2015_s2	92	Senior	mf	AUS, CAN	Multiple sports	competition level	questionnaire	group comp	publ
46	Hopwood et al., 2015_s3	93	Senior	mf	AUS, CAN	Multiple sports	competition level	questionnaire	group comp	publ
47	Hornig et al., 2016_s1	94	Senior	m	GER	Soccer	competition level	questionnaire	group comp	publ
47	Hornig et al., 2016_s2	95	Senior	m	GER	Soccer	competition level	questionnaire	group comp	publ
47	Hornig et al., 2016_s3	96	Senior	m	GER	Soccer	competition level	questionnaire	group comp	publ
48	Huijgen et al., 2009	97	Senior	m	NED	Soccer	coach evaluation	n.a.	group comp	publ
49	Huijgen et al., 2013	98	Junior	m	NED	Soccer	coach evaluation	n.a.	group comp	publ
50	Hutchinson et al., 2013	99	Junior	mf	USA, RUS, MEX	Ballet	coach evaluation	questionnaire	correl	publ
51	Johnson et al., 2006	100	Senior	mf	USA	Swimming	competition level	interview	group comp	publ
52	Lemez et al., 2020	101	Senior	mf	CAN	Wheelchair basketball	competition level	questionnaire	group comp	publ
53	Low et al., 2017	102	Senior	m	MAL	Badminton	competition level	questionnaire	group comp	publ
54	Maynard et al., 2016	103	Senior	mf	USA	Bowling	competition level	interview	correl	unp
55	Memmert et al., 2010_s1	104	Senior	mf	GER	Game sports: BB, FH, SO	competition level	questionnaire	group comp	publ
55	Memmert et al., 2010_s2	105	Senior	mf	GER	Game sports: BB, FH, SO	competition level	questionnaire	group comp	publ
55	Memmert et al., 2010_s3	106	Senior	mf	GER	Game sports: BB, FH, SO	competition level	questionnaire	group comp	publ
56	Mendes et al., 2018	107	Junior	m	BRA	Volleyball	coach evaluation	questionnaire	group comp	publ
57	Moesch et al., 2011	108	Senior	mf	DEN	Cgs sports	competition level	questionnaire	group comp	publ
58	Moseid et al.,2019	109	Junior	mf	NOR	Multiple sports	coach evaluation	questionnaire	group comp	publ
59	Noon et al., 2019	110	Junior	m	GBR	Soccer	coach evaluation	docu-analysis	group comp	publ
60	O'Connor et al., 2016	111	Junior	m	AUS	Soccer	coach evaluation	questionnaire	group comp	publ
61	Roca et al., 2012_s1	112	Senior	m	GBR	Soccer	lab testing	questionnaire	group comp	publ
61	Roca et al., 2012_s2	113	Senior	m	GBR	Soccer	lab testing	questionnaire	group comp	publ
61	Roca et al., 2012_s3	114	Senior	m	GBR	Soccer	lab testing	questionnaire	group comp	publ

Ref. N°	Reference	Study N°	Age_Cat	Sex	Country	Sports [a]	Method definition performance	Method data collection	Method data analysis	Published
62	San & Lee, 2014	115	Junior	mf	MAL	Swimming	competition level	questionnaire	group comp	publ
63	Sawai et al., 2018	116	Senior	f	JAP	Multiple sports	competition level	questionnaire	group comp	publ
64	Sigmund & Güllich, 2020_s1	117	Senior	mf	GER	Athletics, volleyball	competition level	questionnaire	group comp	unp
64	Sigmund & Güllich, 2020_s2	118	Junior	mf	GER	Athletics, volleyball	competition level	questionnaire	group comp	unp
65	Smith, 2012	119	Senior	f	USA	Basketball	competition level	questionnaire	group comp	unp
66	Toering et al., 2009	120	Junior	m	NED	Soccer	competition level	questionnaire	group comp	publ
67	Urena, 2004	121	Junior	f	USA, MEX, RUS	Ballet	coach evaluation	questionnaire	correl	unp
68	Ward et al., 2007	122	Junior	m	GBR	Soccer	competition level	questionnaire	group comp	publ
69	Weissensteiner et al., 2008_s1	123	Senior	m	AUS	Cricket (batsmen)	coach evaluation	interview	group comp	publ
69	Weissensteiner et al., 2008_s2	124	Junior	m	AUS	Cricket (batsmen)	coach evaluation	interview	group comp	publ
69	Weissensteiner et al., 2008_s3	125	Junior	m	AUS	Cricket (batsmen)	coach evaluation	interview	group comp	publ
70	Williams et al., 2012_s1	126	Junior	m	GBR	Soccer	competition level	questionnaire	correl	publ
70	Williams et al., 2012_s2	127	Junior	m	GBR	Soccer	competition level	questionnaire	group comp	publ
70	Williams et al., 2012_s3	128	Junior	m	GBR	Soccer	competition level	questionnaire	group comp	publ
71	Young & Salmela, 2010_s1	129	Junior	m	CAN	Athletics (middle distance)	competition level	questionnaire	group comp	publ
71	Young & Salmela, 2010_s2	130	Junior	m	CAN	Athletics (middle distance)	competition level	questionnaire	group comp	publ
71	Young & Salmela, 2010_s3	131	Junior	m	CAN	Athletics (middle distance)	competition level	questionnaire	group comp	publ

[a] BB = basketball, FH = field hockey, NB = netball, SO = soccer

Ref. N°	Reference	Study N°	Performance level high [b]	HP n	Performance level low [b]	LP n	N	Bandwidth	d perf milestones[c]	d MSPr start	d MSPr	d MSPl	d OSPrPl	d OSPr	d OSPl
1	Baker et al., 2003a	1	WCl	15	RCl_bRCl	13	28	extreme			1.079				
2	Baker et al., 2003b	2	WCl	15	RCl_bRCl	13	28	extreme			1.140	-0.540			
3	Baker et al., 2005_s1	3	bRCl_high	9	bRCl_low	9	18	narrow			0.755		0.321		
3	Baker et al., 2005_s2	4	NCl	9	bRCl_high	9	18	medium			1.926		0.913		
3	Baker et al., 2005_s3	5	NCl	9	bRCl_low	9	18	extreme			2.977		1.236		
4	Baker et al., 2006_s1	6	bRCl_high	6	bRCl_low	6	12	narrow			0.236		0.312		
4	Baker et al., 2006_s2	7	NCl	6	bRCl_high	6	12	medium			2.595		0.808		
4	Baker et al., 2006_s3	8	NCl	6	bRCl_low	6	12	extreme			2.260		1.044		
5	Baker et al., 2012_s1	9	NCl_high	19	NCl_low	24	43	narrow			0.187		0.322		
5	Baker et al., 2012_s2	10	NCl_high	46	NCl_low	40	86	narrow			-0.186		0.513		
6	Barreiros et al., 2013	11	NCl_high	21	NCl_low_RCl	21	42	medium	3.396	0.069	-0.036	-0.017	0.332	0.323	0.340
7	Berry et al., 2008	12	NCl_high	17	NCl_low	15	32	narrow	-0.412	-0.051	0.620	1.060	0.415	0.470	0.360
8	Bjoerndal et al., 2016_s1	13	NCl	21	RCl_high	6	27	narrow			0.634				
8	Bjoerndal et al., 2016_s2	14	NCl	21	RCl_low	6	27	medium			4.984				
8	Bjoerndal et al., 2016_s3	15	RCl_high	6	RCl_low	6	12	narrow			2.215				
9	Bruce et al., 2013	16	WCl	19	bRCl	18	37	extreme		1.082	1.429		-0.055	-0.145	0.036
10	Casado et al., 2019_s1	17	WCl	19	NCl_high	18	37	narrow		0.889					
10	Casado et al., 2019_s2	18	WCl	19	NCl_low	18	37	narrow		1.860					
10	Casado et al., 2019_s3	19	NCl_high	18	NCl_low	18	36	narrow		0.870					
11	Cathey & French, 2014	20	RCl	9	bRCl	9	18	narrow			0.889				
12	Cathey, 2010	21	RCl	11	bRCl	10	21	narrow		0.851	0.521				
13	Coelho-e-Silva et al., 2012	22	WCl	32	RCl	41	73	medium			0.682				
14	Coutinho et al., 2014	23	NCl	140	RCl	89	229	narrow		0.275	0.647		0.068		
15	Coutinho et al., 2015_s1	24	NCl	15	RCl	15	30	narrow		0.000	1.243		2.476	2.476	
15	Coutinho et al., 2015_s2	25	NCl	15	RCl	15	30	narrow		0.297	1.017		0.280	0.280	
16	Coutinho et al., 2016_s1	26	NCl	15	bRCl	15	30	extreme					0.445	0.734	0.155
16	Coutinho et al., 2016_s2	27	NCl	15	bRCl	15	30	extreme					-0.051	0.054	-0.156
17	Da Matta, 2004	28	WCl	10	bRCl	10	20	extreme		0.162	1.183				
18	Dehghansai et al., 2016	29	NCl_high	22	NCL_low	26	48	narrow			0.850				
19	Drake & Breslin, 2017	30	NCl_high	11	NCl_low	12	23	narrow	-1.160	-0.513	0.772	0.492	0.268		
20	Duffy et al., 2004_s1	31	WCl	12	bRCl	12	24	extreme			1.348	0.142			
20	Duffy et al., 2004_s2	32	WCl	6	bRCl	6	12	extreme			1.526	1.817			
21	Elferink-Gemser et al., 2004_s1	33	NCl_high	17	NCl_low	46	63	narrow			0.965				
21	Elferink-Gemser et al., 2004_s2	34	NCl_high	21	NCl_low	42	63	narrow			1.523				
22	Elferink-Gemser et al., 2006_s1	35	NCl_high	15	NCl_low	18	33	narrow			0.512				
22	Elferink-Gemser et al., 2006_s2	36	NCl_high	15	NCl_low	17	32	narrow			1.179				
23	Elferink-Gemser et al., 2011_s1	37	NCl_high	31	NCl_low	66	97	narrow			0.290				
23	Elferink-Gemser et al., 2011_s2	38	NCl_high	32	NCl_low	95	127	narrow			0.450				

Ref. N°	Reference	Study N°	Performance level high [b]	HP n	Performance level low [b]	LP n	N	Bandwidth	d perf milestones[c]	d MSPr start	d MSPr	d MSPl	d OSPrPl	d OSPr	d OSPl
24	Erikstad et al., 2018	39	NCl	105	RCl	410	515	narrow			0.088	-0.004			
25	Ford & Williams, 2012	40	NCl	16	RCl	16	32	narrow	-0.180	-0.615	0.455	0.645	0.063		
26	Ford et al., 2009_s1	41	NCl_high	11	NCl_low	11	22	narrow	0.300	-0.107	-0.400	0.800	-0.300		
26	Ford et al., 2009_s2	42	NCl	22	bRCl	11	33	extreme		0.000	1.500	0.400	-0.700		
27	Ford et al., 2010	43	NCl_high	15	NCl_low	15	30	narrow	0.210	-0.015	0.870	0.603	0.430		
28	Guimaraes et al., 2019	44	NCl	75	RCl	75	150	narrow			0.799				
29	Güllich & Emrich, 2014_s1	45	WCl	208	NCl	97	305	narrow	0.287	0.303	-0.011		0.193	0.319	0.067
29	Güllich & Emrich, 2014_s10	46	WCl_NCl_high	46	NCl_low	75	121	medium	-0.882	-0.823	0.712		-0.125	0.093	-0.343
29	Güllich & Emrich, 2014_s11	47	WCl	20	NCl	314	334	narrow	-0.603	-0.254	0.273		-0.089	-0.165	-0.012
29	Güllich & Emrich, 2014_s12	48	WCl	18	NCl	148	166	narrow	-0.899	-0.095	0.802		-0.199	-0.189	-0.210
29	Güllich & Emrich, 2014_s13	49	WCl	7	NCl	86	93	narrow	-0.320	0.516	-0.256		-0.037	-0.108	0.033
29	Güllich & Emrich, 2014_s14	50	WCl	16	NCl	75	91	narrow	-0.607	-0.348	-0.041		-0.351	-0.236	-0.466
29	Güllich & Emrich, 2014_s15	51	WCl	8	NCl	62	70	narrow	-0.441	-0.348	1.123		-0.304	-0.291	-0.318
29	Güllich & Emrich, 2014_s2	52	WCl	73	NCl	44	117	narrow	0.013	0.138	-0.350		0.340	0.380	0.299
29	Güllich & Emrich, 2014_s3	53	WCl	40	NCl	24	64	narrow	0.480	0.475	-0.088		0.344	0.362	0.326
29	Güllich & Emrich, 2014_s4	54	WCl	20	NCl	24	44	narrow	0.940	0.399	0.396		0.094	0.331	-0.143
29	Güllich & Emrich, 2014_s6	55	WCl_NCl_high	220	NCl_low	337	557	medium	-0.689	-0.933	1.116		-0.252	-0.309	-0.196
29	Güllich & Emrich, 2014_s7	56	WCl_NCl_high	91	NCl_low	139	230	medium	-0.698	-0.511	0.425		-0.180	-0.215	-0.144
29	Güllich & Emrich, 2014_s8	57	WCl_NCl_high	59	NCl_low	51	110	medium	-0.578	-0.516	0.443		-0.202	-0.217	-0.188
29	Güllich & Emrich, 2014_s9	58	WCl_NCl_high	77	NCl_low	20	97	medium	-0.254	-0.523	0.277		-0.233	-0.551	0.084
30	Güllich et al., 2020	59	RCl_high	50	RCl_low	50	100	narrow	-0.215	-0.318	0.916	0.161	-0.029	-0.140	0.082
31	Güllich, 2014	60	WCl	16	NCl	19	35	narrow	0.830	-0.365	-0.523	-0.099	0.613	0.755	0.470
32	Güllich, 2017_s1	61	WCl	44	NCl	44	88	narrow	0.286	0.542	-0.885		0.524	0.878	0.169
32	Güllich, 2017_s2	62	WCl	12	NCl	12	24	narrow	-0.095	0.232	-0.769		0.525	0.506	0.543
32	Güllich, 2017_s3	63	WCl	8	NCl	8	16	narrow	0.000	1.196	-1.614		0.675	1.619	-0.270
32	Güllich, 2017_s4	64	WCl	5	NCl	5	10	narrow	0.230	1.355	-0.845		0.149	0.740	-0.443
32	Güllich, 2017_s5	65	WCl	14	NCl	14	28	narrow	-0.060	0.120	-0.961		0.616	1.244	-0.012
33	Güllich, 2018a_s1	66	WCl_NCl_high	40	NCl_low	40	80	narrow	1.304	0.349	-0.209	-0.371	0.471	0.876	0.009
33	Güllich, 2018a_s2	67	WCl	17	NCl	17	34	narrow	1.493	0.440	-0.168	-0.671	0.445	1.154	-0.364
33	Güllich, 2018a_s3	68	WCl_NCl_high	69	NCl_low	69	138	narrow	-0.297	0.083	0.355	-0.023	0.072	0.090	0.051
33	Güllich, 2018a_s4	69	WCl	23	NCl	23	46	narrow	-0.707	0.084	0.415	0.028	0.005	-0.008	0.020
34	Güllich, 2019	70	WCl	14	NCl	15	29	narrow	0.884	0.708	-0.619	0.818	0.399	0.842	-0.017
35	Gunn et al., 2019	71	NCl	13	RCl	17	30	narrow			-0.654		0.407	0.407	
36	Hardy et al., 2013	72	WCl	16	NCl	16	32	narrow	0.852	0.510	0.323	0.033	0.341	0.282	0.400
37	Harris, 2008	73	RCl	16	bRCl	16	32	narrow		0.319	0.904	0.633			
38	Haugaasen et al., 2014	74	NCl	81	RCl	462	543	narrow		-0.060	0.240				
39	Helsen et al., 1998_s1	75	NCl	17	RCl_high	21	38	narrow			0.743				
39	Helsen et al., 1998_s2	76	RCl_high	21	RCl_low	35	56	narrow			1.059				

Ref. N°	Reference	Study N°	Performance level high [b]	HP n	Performance level low [b]	LP n	N	Bandwidth	d perf milestones[c]	d MSPr start	d MSPr	d MSPl	d OSPrPl	d OSPr	d OSPl
39	Helsen et al., 1998_s3	77	NCl	17	RCl_low	35	52	extreme			1.731				
39	Helsen et al., 1998_s4	78	NCl_high	16	NCl_low	18	34	narrow			-0.055				
39	Helsen et al., 1998_s5	79	NCl_low	18	RCl	17	35	narrow			0.792				
39	Helsen et al., 1998_s6	80	NCl_high	16	RCl	17	33	extreme			0.804				
40	Hendry & Hodges, 2018_s1	81	NCl	9	RCl	17	26	narrow	-0.070	-0.180	-0.124	2.020	0.047		
40	Hendry & Hodges, 2018_s2	82	NCl	26	RCl	76	102	narrow	-1.040	-0.230	0.600	0.190	-0.150		
41	Hendry et al., 2018_s1	83	NCl	9	RCl	17	26	narrow			0.673	0.282			
41	Hendry et al., 2018_s2	84	NCl	26	RCl	76	102	narrow			0.018	-0.133			
42	Hendry et al., 2019a	85	NCl	31	RCl	32	63	narrow		-0.337	1.205	1.730	-1.430		
43	Hendry et al., 2019b	86	WCl	21	RCl	24	45	medium	0.739	0.780	0.410	1.463	0.273		
44	Hendry, 2012_s1	87	NCl_h_to_NCl_low	48			48	narrow			0.814	-0.341			
44	Hendry, 2012_s2	88	NCl_h_to_NCl_low	50			50	narrow			0.385	0.064			
44	Hendry, 2012_s3	89	NCl_h_to_NCl_low	46			46	narrow			0.108	0.042			
45	Hodges et al., 2015	90	RCl_to_bRCl	90			90	narrow			0.539				
46	Hopwood et al., 2015_s1	91	NCl_high	122	NCl_low	32	154	narrow		-0.001					
46	Hopwood et al., 2015_s2	92	NCl_low	32	RCl_bRCl	55	87	medium		0.047					
46	Hopwood et al., 2015_s3	93	NCl_high	122	RCl_bRCl	55	177	extreme		0.047					
47	Hornig et al., 2016_s1	94	WCl	18	NCl	34	52	narrow	0.259	-0.524	-0.266	0.038	0.272	0.386	0.158
47	Hornig et al., 2016_s2	95	NCl	34	RCl	50	84	narrow	0.544	0.215	0.516	0.099	-0.048	-0.049	-0.046
47	Hornig et al., 2016_s3	96	WCl	18	RCl	50	68	extreme	0.937	-0.290	0.295	0.155	0.227	0.348	0.105
48	Huijgen et al., 2009	97	NCl	11	RCl	15	26	narrow			0.649				
49	Huijgen et al., 2013	98	NCl	76	RCl	37	113	narrow	-0.448	0.124	0.240				
50	Hutchinson et al., 2013	99	NCl_to_bRCl	47			47	extreme		-0.020	0.926				
51	Johnson et al., 2006	100	WCl	7	NCl	9	16	narrow	-0.424	0.434	-0.129		0.786	0.786	
52	Lemez et al., 2020	101	WCl	37	NCl	21	58	narrow					0.256	0.256	
53	Low et al., 2017	102	NCl	16	RCl	20	36	narrow	0.721	1.208	-0.700	-0.996			
54	Maynard et al., 2016	103	bRCl	92			92	medium			0.676	-0.499			
55	Memmert et al., 2010_s1	104	NCl	24	RCl_high	24	48	narrow		0.682	0.321	-0.636	0.089		
55	Memmert et al., 2010_s2	105	RCl_high	24	RCl_low	24	48	narrow		-0.168	-0.688	-0.468	-0.043		
55	Memmert et al., 2010_s3	106	NCl	24	RCl_low	24	48	medium		0.528	-0.281	-1.103	0.041		
56	Mendes et al., 2018	107	NCl_high	14	NCl_low	64	78	narrow		-0.519	-0.129		-0.401	-0.401	
57	Moesch et al., 2011	108	WCl	143	NCl	94	237	narrow	0.420	0.630	-0.230		0.060		
58	Moseid et al.,2019	109	NCl_high	96	NCl_low	114	210	narrow					-0.260	-0.260	
59	Noon et al., 2019	110	NCl	16	RCl	60	76	narrow	-0.504						
60	O'Connor et al., 2016	111	NCl	22	RCl	105	127	narrow			0.057	-0.259			
61	Roca et al., 2012_s1	112	RCl_high	16	RCl_low	16	32	narrow	0.362	-0.224	0.267	0.670	0.074		
61	Roca et al., 2012_s2	113	RCl_low	16	bRCl	16	32	narrow		-0.099	1.317	0.603	-0.071		
61	Roca et al., 2012_s3	114	RCl_high	16	bRCl	16	32	medium		-0.343	1.398	1.194	-0.002		

Ref. Nº	Reference	Study Nº	Performance level high [b]	HP n	Performance level low [b]	LP n	N	Bandwidth	d perf milestones[c]	d MSPr start	d MSPr	d MSPl	d OSPrPl	d OSPr	d OSPl
62	San & Lee, 2014	115	NCl	15	RCl	15	30	medium		-0.128	1.145	0.155	-2.327		
63	Sawai et al., 2018	116	NCl	258	RCl_bRCl	273	531	medium		-0.300	0.492				
64	Sigmund & Güllich, 2020_s1	117	WCl	72	NCl	116	188	narrow			-0.087	-0.093	0.292	0.526	0.057
64	Sigmund & Güllich, 2020_s2	118	WCl	67	NCl	172	239	narrow			0.447	-0.067	-0.045	-0.117	-0.018
65	Smith, 2012	119	RCl_high	36	RCl_low	36	72	narrow		0.506	-0.510	-0.321			
66	Toering et al., 2009	120	NCl	159	RCl_bRCl	285	444	medium			3.228		-0.460	-0.460	
67	Urena, 2004	121	NCl_to_bRCl	195			195	extreme			0.985				
68	Ward et al., 2007	122	NCl	102	bRCl	101	203	extreme		-0.623	4.782	-1.098	-1.190	-1.003	-1.376
69	Weissensteiner et al., 2008_s1	123	NCl	13	bRCl	10	23	extreme			1.482	-0.297	-0.024	-0.207	0.160
69	Weissensteiner et al., 2008_s2	124	NCl	18	bRCl	20	38	extreme			0.708	-0.322	0.063	0.186	-0.061
69	Weissensteiner et al., 2008_s3	125	NCl	21	bRCl	20	41	extreme			1.206	0.810	0.312	0.224	0.400
70	Williams et al., 2012_s1	126	NCl	12	RCl	12	24	medium				0.680			
70	Williams et al., 2012_s2	127	NCl	12	bRCl	12	24	extreme				1.634			
70	Williams et al., 2012_s3	128	RCl	12	bRCl	12	24	medium				1.029			
71	Young & Salmela, 2010_s1	129	NCl	10	RCl	24	34	narrow		0.667	0.394				
71	Young & Salmela, 2010_s2	130	RCl	24	bRCl	14	38	narrow		-0.378	0.680				
71	Young & Salmela, 2010_s3	131	NCl	10	bRCl	14	24	extreme		0.215	0.977				

[b] WCl = world class, NCl = national class,
RCl = regional class, bRCl = below regional class

[c] d = Cohen's *d* , MS = main sport, OS = other sports,
Pr = practice, Pl = play

Ref. N°	Reference	Study N°	Source of Cohen's d	Annotation
1	Baker et al., 2003a	1	converted from reported F	HP: coach evaluation, LP: competition level
2	Baker et al., 2003b	2	reported d	HP: coach evaluation, LP: competition level
3	Baker et al., 2005_s1	3	calculated from reported M ± SD	
3	Baker et al., 2005_s2	4	calculated from reported M ± SD	
3	Baker et al., 2005_s3	5	calculated from reported M ± SD	
4	Baker et al., 2006_s1	6	calculated from reported M ± SD	
4	Baker et al., 2006_s2	7	calculated from reported M ± SD	
4	Baker et al., 2006_s3	8	calculated from reported M ± SD	
5	Baker et al., 2012_s1	9	calculated from reported M ± SD (partly from figure)	
5	Baker et al., 2012_s2	10	calculated from reported M ± SD (partly from figure)	
6	Barreiros et al., 2013	11	calculated from reported M ± SD	sex controlled for
7	Berry et al., 2008	12	reported d or calculated from reported M ± SD	
8	Bjoerndal et al., 2016_s1	13	calculated from reported M ± SD	
8	Bjoerndal et al., 2016_s2	14	calculated from reported M ± SD	
8	Bjoerndal et al., 2016_s3	15	calculated from reported M ± SD	
9	Bruce et al., 2013	16	calculated from reported M ± SD	HP: coach evaluation, LP: competition level
10	Casado et al., 2019_s1	17	calculated from reported M ± SD	
10	Casado et al., 2019_s2	18	calculated from reported M ± SD	
10	Casado et al., 2019_s3	19	calculated from reported M ± SD	
11	Cathey & French, 2014	20	calculated from reported M ± SD	
12	Cathey, 2010	21	calculated from reported M ± SD	
13	Coelho-e-Silva et al., 2012	22	calculated from reported M ± SD	
14	Coutinho et al., 2014	23	converted from reported χ^2 (frequencies within categories) or F	sex controlled for
15	Coutinho et al., 2015_s1	24	calculated from reported M ± SD	(d)
15	Coutinho et al., 2015_s2	25	calculated from reported M ± SD	(d)
16	Coutinho et al., 2016_s1	26	calculated from reported M ± SD	
16	Coutinho et al., 2016_s2	27	calculated from reported M ± SD	
17	Da Matta, 2004	28	calculated from reported M ± SD or converted from reported F	HP: coach evaluation, LP: competition level
18	Dehghansai et al., 2016	29	reported d	
19	Drake & Breslin, 2017	30	reported d or calculated from reported M ± SD (partly from figure)	(d)
20	Duffy et al., 2004_s1	31	calculated from reported M ± SD	
20	Duffy et al., 2004_s2	32	calculated from reported M ± SD	
21	Elferink-Gemser et al., 2004_s1	33	calculated from reported M ± SD	
21	Elferink-Gemser et al., 2004_s2	34	calculated from reported M ± SD	
22	Elferink-Gemser et al., 2006_s1	35	calculated from reported M ± SD	
22	Elferink-Gemser et al., 2006_s2	36	calculated from reported M ± SD	
23	Elferink-Gemser et al., 2011_s1	37	reported d	sex controlled for
23	Elferink-Gemser et al., 2011_s2	38	reported d	sex controlled for

Ref. N°	Reference	Study N°	Source of Cohen's d	Annotation
24	Erikstad et al., 2018	39	converted from reported r_s	
25	Ford & Williams, 2012	40	reported d	
26	Ford et al., 2009_s1	41	reported d or calculated from reported M ± SD	
26	Ford et al., 2009_s2	42	reported d or calculated from reported M ± SD	HP: coach evaluation, LP: competition level
27	Ford et al., 2010	43	reported d	
28	Guimaraes et al., 2019	44	converted from reported r	
29	Güllich & Emrich, 2014_s1	45	reported d and raw data	sex controlled for; OSPI unpublished[(d)]
29	Güllich & Emrich, 2014_s10	46	reported d and raw data	sex controlled for[(d)]
29	Güllich & Emrich, 2014_s11	47	reported d and raw data	sex controlled for[(d)]
29	Güllich & Emrich, 2014_s12	48	reported d and raw data	sex controlled for[(d)]
29	Güllich & Emrich, 2014_s13	49	reported d and raw data	sex controlled for[(d)]
29	Güllich & Emrich, 2014_s14	50	reported d and raw data	sex controlled for[(d)]
29	Güllich & Emrich, 2014_s15	51	reported d and raw data	sex controlled for[(d)]
29	Güllich & Emrich, 2014_s2	52	reported d and raw data	sex controlled for; OSPI unpublished[(d)]
29	Güllich & Emrich, 2014_s3	53	reported d and raw data	sex controlled for; OSPI unpublished[(d)]
29	Güllich & Emrich, 2014_s4	54	reported d and raw data	sex controlled for; OSPI unpublished[(d)]
29	Güllich & Emrich, 2014_s6	55	reported d and raw data	sex controlled for; OSPI unpublished[(d)]
29	Güllich & Emrich, 2014_s7	56	reported d and raw data	sex controlled for; OSPI unpublished[(d)]
29	Güllich & Emrich, 2014_s8	57	reported d and raw data	sex controlled for; OSPI unpublished[(d)]
29	Güllich & Emrich, 2014_s9	58	reported d and raw data	sex controlled for; OSPI unpublished[(d)]
30	Güllich et al., 2020	59	converted from raw data	[(d)]
31	Güllich, 2014	60	reported d and raw data	[(d)]
32	Güllich, 2017_s1	61	reported d and raw data	matched pairs; OSPI unpublished[(d)]
32	Güllich, 2017_s2	62	reported d and raw data	matched pairs; OSPI unpublished[(d)]
32	Güllich, 2017_s3	63	reported d and raw data	matched pairs; OSPI unpublished[(d)]
32	Güllich, 2017_s4	64	reported d and raw data	matched pairs; OSPI unpublished[(d)]
32	Güllich, 2017_s5	65	reported d and raw data	matched pairs; OSPI unpublished[(d)]
33	Güllich, 2018a_s1	66	reported d and raw data	matched pairs[(d)]
33	Güllich, 2018a_s2	67	reported d and raw data	matched pairs[(d)]
33	Güllich, 2018a_s3	68	reported d and raw data	matched pairs[(d)]
33	Güllich, 2018a_s4	69	reported d and raw data	matched pairs[(d)]
34	Güllich, 2019	70	reported d and raw data	[(d)]
35	Gunn et al., 2019	71	converted from reported r	
36	Hardy et al., 2013	72	caluclated from reported M ± SD and raw data	matched pairs[(d)]
37	Harris, 2008	73	calculated from reported M ± SD	
38	Haugaasen et al., 2014	74	from reported ES, p	
39	Helsen et al., 1998_s1	75	calculated from reported M ± SD (partly from figure)	
39	Helsen et al., 1998_s2	76	calculated from reported M ± SD (partly from figure)	

Ref. N°	Reference	Study N°	Source of Cohen's d	Annotation
39	Helsen et al., 1998_s3	77	calculated from reported M ± SD (partly from figure)	
39	Helsen et al., 1998_s4	78	calculated from reported M ± SD (partly from figure)	HP: coach evaluation, LP: competition level
39	Helsen et al., 1998_s5	79	calculated from reported M ± SD (partly from figure)	
39	Helsen et al., 1998_s6	80	calculated from reported M ± SD (partly from figure)	HP: coach evaluation, LP: competition level
40	Hendry & Hodges, 2018_s1	81	reported d	
40	Hendry & Hodges, 2018_s2	82	reported d	
41	Hendry et al., 2018_s1	83	converted from reported r	
41	Hendry et al., 2018_s2	84	converted from reported r	
42	Hendry et al., 2019a	85	reported d	
43	Hendry et al., 2019b	86	calculated from reported M ± SD (partly from figure)	
44	Hendry, 2012_s1	87	converted from reported r	
44	Hendry, 2012_s2	88	converted from reported r	
44	Hendry, 2012_s3	89	converted from reported r	
45	Hodges et al., 2015	90	converted from reported r	
46	Hopwood et al., 2015_s1	91	calculated from reported M ± SD	
46	Hopwood et al., 2015_s2	92	calculated from reported M ± SD	
46	Hopwood et al., 2015_s3	93	calculated from reported M ± SD	
47	Hornig et al., 2016_s1	94	reported d and raw data	[d]
47	Hornig et al., 2016_s2	95	reported d and raw data	[d]
47	Hornig et al., 2016_s3	96	reported d and raw data	[d]
48	Huijgen et al., 2009	97	calculated from reported M ± SD	
49	Huijgen et al., 2013	98	calculated from reported M ± SD	
50	Hutchinson et al., 2013	99	converted from reported r	sex controlled for
51	Johnson et al., 2006	100	calculated from reported individual raw data (from figure)	sex controlled for[d]
52	Lemez et al., 2020	101	calculated from reported M ± SD	
53	Low et al., 2017	102	calculated from reported M ± SD	
54	Maynard et al., 2016	103	converted from reported r	[d]
55	Memmert et al., 2010_s1	104	calculated from reported M ± SD and raw data	sex controlled for[d]
55	Memmert et al., 2010_s2	105	calculated from reported M ± SD and raw data	sex controlled for[d]
55	Memmert et al., 2010_s3	106	calculated from reported M ± SD and raw data	sex controlled for[d]
56	Mendes et al., 2018	107	calculated from reported M ± SD	[d]
57	Moesch et al., 2011	108	reported d and raw data	sex controlled for[d]
58	Moseid et al.,2019	109	calculated from reported frequency distributions	
59	Noon et al., 2019	110	calculated from reported frequency distributions	
60	O'Connor et al., 2016	111	calculated from reported M ± SD	
61	Roca et al., 2012_s1	112	calculated from reported M ± SD	
61	Roca et al., 2012_s2	113	calculated from reported M ± SD	HP: lab testing, LP: competition level
61	Roca et al., 2012_s3	114	calculated from reported M ± SD	HP: lab testing, LP: competition level

Ref. N°	Reference	Study N°	Source of Cohen's d	Annotation
62	San & Lee, 2014	115	calculated from reported M ± SD	
63	Sawai et al., 2018	116	calculated from reported M ± SD	
64	Sigmund & Güllich, 2020_s1	117	calculated from raw data	sex controlled for[d]
64	Sigmund & Güllich, 2020_s2	118	calculated from raw data	sex controlled for[d]
65	Smith, 2012	119	converted from reported F or calculated from reported raw data	
66	Toering et al., 2009	120	calculated from reported M ± SD	
67	Urena, 2004	121	converted from reported r or F	
68	Ward et al., 2007	122	calculated from reported M ± SD (partly from figure)	
69	Weissensteiner et al., 2008_s1	123	calculated from reported M ± SD (partly from figure)	HP: coach evaluation, LP: competition level[d]
69	Weissensteiner et al., 2008_s2	124	calculated from reported M ± SD (partly from figure)	HP: coach evaluation, LP: competition level[d]
69	Weissensteiner et al., 2008_s3	125	calculated from reported M ± SD (partly from figure)	HP: coach evaluation, LP: competition level[d]
70	Williams et al., 2012_s1	126	calculated from reported M ± SD	
70	Williams et al., 2012_s2	127	calculated from reported M ± SD	
70	Williams et al., 2012_s3	128	calculated from reported M ± SD	
71	Young & Salmela, 2010_s1	129	calculated from reported M ± SD (partly from figure)	
71	Young & Salmela, 2010_s2	130	calculated from reported M ± SD (partly from figure)	
71	Young & Salmela, 2010_s3	131	calculated from reported M ± SD (partly from figure)	

[d] Direct interaction with authors and/or original data were made available.

1. Baker J, Cote J, Abernethy B. Sport-specific practice and the development of expert decision-making in team ball sports. Journal of Applied Sport Psychology. 2003;15:12–25. doi:10.1080/10413200305400.
2. Baker J, Côté J, Abernethy B. Learning from the experts: practice activities of expert decision makers in sport. Res Q Exerc Sport. 2003;74:342–7. doi:10.1080/02701367.2003.10609101.
3. Baker J, Côté J, Deakin J. Expertise in ultra-endurance triathletes early sport involvement, training structure, and the theory of deliberate practice. J Appl Sport Psychol. 2005;17:64–78. doi:10.1080/10413200590907577.
4. Baker J, Côte J, Deakin J. Patterns of early involvement in expert and nonexpert masters triathletes. Res Q Exerc Sport. 2006;77:401–7. doi:10.1080/02701367.2006.10599375.
5. Baker J, Bagats S, Büsch D, Strauss B, Schorer J. Training differences and selection in a talent identification system. Talent Development & Excellence. 2012;4:23–32.
6. Barreiros A, Côté J, Fonseca AM. Training and psychosocial patterns during the early development of Portuguese national team athletes. High Ability Studies. 2013;24:49–61. doi:10.1080/13598139.2013.780965.
7. Berry J, Abernethy B, Côté J. The contribution of structured activity and deliberate play to the development of expert perceptual and decision-making skill. J Sport Exerc Psychol. 2008;30:685–708. doi:10.1123/jsep.30.6.685.
8. Bjørndal CT, Ronglan LT, Andersen SS. The diversity of developmental paths among youth athletes: a 3-year longitudinal study of Norwegian handball players. Journal of Talent Development and Excellence. 2016;8:20–32.
9. Bruce L, Farrow D, Raynor A. Performance milestones in the development of expertise: are they critical? J Appl Sport Psychol. 2013;25:281–97. doi:10.1080/10413200.2012.725704.

10. Casado A, Hanley B, Ruiz-Pérez LM. Deliberate practice in training differentiates the best Kenyan and Spanish long-distance runners. Eur J Sport Sci. 2019;online first. doi:10.1080/17461391.2019.1694077.

11. Cathey RM, French KE. Retrospective practice histories of expert and novice baseball pitchers. Res Q Exerc Sport. 2014;85:A108. doi:10.1080/02701367.2014.948745.

12. Cathey RM. Retrospective practice histories of expert and novice baseball pitchers [Doctoral Thesis]. Columbia: University of South Carolina; 2010.

13. Coelho-E-Silva MJ, Vaz V, Simões F, Carvalho HM, Valente-Dos-Santos J, Figueiredo AJ, et al. Sport selection in under-17 male roller hockey. J Sports Sci. 2012;30:1793–802. doi:10.1080/02640414.2012.709262.

14. Coutinho P, Mesquita I, Fonseca AM, Martin-Silva L de. Patterns of sport participation in Portuguese volleyball players according to expertise level and gender. Int J Sports Sci Coach. 2014;9:579–92. doi:10.1260/1747-9541.9.4.579.

15. Coutinho P, Mesquita I, Fonseca AM, Côte J. Expertise development in Volleyball: the role of early sport activities and players' age and height. Kinesiology. 2015;47:215–25.

16. Coutinho P, Mesquita I, Davids K, Fonseca AM, Côté J. How structured and unstructured sport activities aid the development of expertise in volleyball players. Psychol Sport Exerc. 2016;25:51–9. doi:10.1016/j.psychsport.2016.04.004.

17. Da Matta GB. The influence of deliberate practice and social support systems of the development of expert and intermediate women volleyball players in Brazil [Doctoral Thesis]. Columbia: University of South Carolina; 2004.

18. Dehghansai N, Srdjan Lemez, Wattie N, Baker J. Talent or physical precocity; Examination of junior, senior and precocious para-athletes' developmental trajectories and training histories; 2016.

19. Drake D, Breslin G. Developmental activities and the acquisition of perceptual-cognitive expertise in international field hockey players. Int J Sports Sci Coach. 2019;online first. doi:10.1177/1747954117711093.

20. Duffy LJ, Baluch B, Ericsson AK. Dart performance as a function of facets of practice amongst professional and amateur men ana women players. Int J Sport Psychol. 2004;3:232–45.

21. Elferink-Gemser MT, Visscher C, Lemmink KAPM, Mulder TW. Relation between multi-dimensional performance characteristics and level of performance in talented youth field hockey players. J Sports Sci. 2004;22:1053–63. doi:10.1080/02640410410001729991.

22. Elferink-Gemser MT, Visscher C, Lemmink KAPM, Mulder T. Multidimensional performance characteristics and standard of performance in talented youth field hockey players: a longitudinal study. J Sports Sci. 2006;online first. doi:10.1080/02640410600719945.

23. Elferink-Gemser MT, Starkes JL, Medic N, Lemmink KAPM, Visscher, C. What discriminates elite and sub-elite youth field hockey players. Annals of Research in Sport and Physical Activity. 2011:50–68. doi:10.14195/2182-7087_1_3.

24. Erikstad MK, Høigaard R, Johansen BT, Kandala N-B, Haugen T. Childhood football play and practice in relation to self-regulation and national team selection; a study of Norwegian elite youth players. J Sports Sci. 2018;36:2304–10. doi:10.1080/02640414.2018.1449563.

25. Ford PR, Williams AM. The developmental activities engaged in by elite youth soccer players who progressed to professional status compared to those who did not. Psychol Sport Exerc. 2012;13:349–52. doi:10.1016/j.psychsport.2011.09.004.

26. Ford PR, Ward P, Hodges NJ, Williams AM. The role of deliberate practice and play in career progression in sport: the early engagement hypothesis. High Ability Studies. 2009;20:65–75. doi:10.1080/13598130902860721.

27. Ford PR, Low J, McRobert AP, Williams AM. Developmental activities that contribute to high or low performance by elite cricket batters when recognizing type of delivery from bowlers' advanced postural cues. J Sport Exerc Psychol. 2010;32:638–54. doi:10.1123/jsep.32.5.638.

28. Guimarães E, Ramos A, Janeira MA, Baxter-Jones ADG, Maia J. How does biological maturation and training experience impact the physical and technical performance of 11-14-year-old male basketball players? Sports (Basel) 2019. doi:10.3390/sports7120243.

29. Güllich A, Emrich E. Considering long-term sustainability in the development of world class success. Eur J Sport Sci. 2014;14 Suppl 1:S383-S397. doi:10.1080/17461391.2012.706320.

30. Güllich A, Cronauer R, Diehl J, Gard L, Miller C. Coach-assessed skill learning progress of youth soccer players correlates with earlier childhood practice in other sports. Int J Sports Sci Coach. 2020;15:285–96. doi:10.1177/1747954120912351.

31. Güllich A. Many roads lead to Rome—developmental paths to Olympic gold in men's field hockey. Eur J Sport Sci. 2014;14:763–71. doi:10.1080/17461391.2014.905983.

32. Güllich A. International medallists' and non-medallists' developmental sport activities - a matched-pairs analysis. J Sports Sci. 2017;35:2281–8. doi:10.1080/02640414.2016.1265662.

33. Güllich A. Sport-specific and non-specific practice of strong and weak responders in junior and senior elite athletics - a matched-pairs analysis. J Sports Sci. 2018;36:2256–64. doi:10.1080/02640414.2018.1449089.

34. Güllich A. "Macro-structure" of developmental participation histories and "micro-structure" of practice of German female world-class and national-class football players. J Sports Sci. 2019;37:1347–55. doi:10.1080/02640414.2018.1558744.

35. Gunn SM, Mangine GT, VanDusseldorp TA, Hester GM. Influence of sport specialization on athletic performance and injury risk in collegiate swimmers. The Kennesaw Journal of Undergraduate Research. 2019;6:Article 3. doi:10.32727/25.2019.31.

36. Hardy L, Laing S, Barlow M, Kincheva L, Evans L, Rees T, et al. A comparison of the biographies of GB serial medal and non-medalling olympic athletes. London; 2013.

37. Harris KR. Deliberate practice, mental representations, and skilled performance in bowling [Doctoral Thesis]. Tallahassee: Florida State University; 2008.

38. Haugaasen M, Toering T, Jordet G. From childhood to senior professional football: a multi-level approach to elite youth football players' engagement in football-specific activities. Psychol Sport Exerc. 2014;15:336–44. doi:10.1016/j.psychsport.2014.02.007.

39. Helsen WF, Starkes JL, Hodges NJ. Team sports and the theory of deliberate practice. J Sport Exerc Psychol. 1998;20:12–34.

40. Hendry DT, Hodges NJ. Early majority engagement pathway best defines transitions from youth to adult elite men's soccer in the UK: a three time-point retrospective and prospective study. Psychol Sport Exerc. 2018;36:81–9. doi:10.1016/j.psychsport.2018.01.009.

41. Hendry DT, Williams AM, Hodges NJ. Coach ratings of skills and their relations to practice, play and successful transitions from youth-elite to adult-professional status in soccer. J Sports Sci. 2018;36:2009–17. doi:10.1080/02640414.2018.1432236.

42. Hendry DT, Crocker PRE, Williams AM, Hodges NJ. Tracking and comparing self-determined motivation in elite youth soccer: influence of developmental activities, age, and skill. Front Psychol. 2019;10:304. doi:10.3389/fpsyg.2019.00304.

43. Hendry DT, Williams AM, Ford PR, Hodges NJ. Developmental activities and perceptions of challenge for National and Varsity women soccer players in Canada. Psychol Sport Exerc. 2019;43:210–8. doi:10.1016/j.psychsport.2019.02.008.

44. Hendry DT. The role of developmental activities on self determined motivation, passion and skill in youth soccer players [Master Thesis]. Vancouver: The University of British Columbia; 2012.

45. Hodges NJ, Augaitis L, Crocker PRE. Sport commitment and deliberate practice among male and female triathletes. Int J Sport Psychol. 2015;46:652–65. doi:10.7352/IJSP.2015.46.652.

46. Hopwood M, Macmahon C, Farrow D, Baker J. Is practice the only determinant of sporting expertise? Revisiting Starkes (2000). Int J Sport Psychol. 2015;46:631–51. doi:10.7352/IJSP.2015.46.631.

47. Hornig M, Aust F, Güllich A. Practice and play in the development of German top-level professional football players. Eur J Sport Sci. 2016;16:96–105. doi:10.1080/17461391.2014.982204.

48. Huijgen BCH, Elferink-Gemser MT, Post WJ, Visscher C. Soccer skill development in professionals. Int J Sports Med. 2009;30:585–91. doi:10.1055/s-0029-1202354.

49. Huijgen BCH, Elfernik-Gemser MT, Post WJ, Visscher C. Soccer skill development in professionals. In: Huijgen BCH, editor. Technical skills the key to success? A study on talent development and selection of youth soccer players [Doctoral Thesis]. Groningen: University of Groningen; 2013. pp. 53–67

50. Hutchinson CU, Sachs-Ericsson NJ, Ericsson KA. Generalizable aspects of the development of expertise in ballet across countries and cultures: a perspective from the expert-performance approach. High Ability Studies. 2013;24:21–47. doi:10.1080/13598139.2013.780966.

51. Johnson MB, Tenenbaum G, Edmonds WA. Adaptation to physically and emotionally demanding conditions: the role of deliberate practice. High Ability Studies. 2006;17:117–36. doi:10.1080/13598130600947184.

52. Lemez S, Wattie N, Dehghansai N, Baker J. Developmental pathways of Para athletes: examining the sporting backgrounds of elite Canadian wheelchair basketball players. CISS. 2020;5:2. doi:10.15203/CISS_2020.002.

53. Low J, Mohamad NI, Ong KB, Aziz SA, Abdullah MR, Maliki A. The developmental pathways of Malaysian elite youth badminton players. Journal of Fundamental and Applied Sciences. 2017;9:842–57. doi:10.4314/jfas.v9i2s.60.

54. Maynard D, Hambrick DZ, Meinz EJ. How important is deliberate practice for explaining expertise in sports? The case of bowling; 2016.

55. Memmert D, Baker J, Bertsch C. Play and practice in the development of sport-specific creativity in team ball sports. High Ability Studies. 2010;21:3–18. doi:10.1080/13598139.2010.488083.

56. Mendes FG, Nascimento JV, Souza ER, Collet C, Milistetd M, Côté J, Carvalho HM. Retrospective analysis of accumulated structured practice: a Bayesian multilevel analysis of elite Brazilian volleyball players. High Ability Studies. 2018;29:255–69. doi:10.1080/13598139.2018.1507901.

57. Moesch K, Elbe A-M, Hauge M-LT, Wikman JM. Late specialization: the key to success in centimeters, grams, or seconds (cgs) sports. Scand J Med Sci Sports. 2011;21:e282-90. doi:10.1111/j.1600-0838.2010.01280.x.

58. Moseid CH, Myklebust G, Fagerland MW, Bahr R. The association between early specialization and performance level with injury and illness risk in youth elite athletes. Scand J Med Sci Sports. 2019;29:460–8. doi:10.1111/sms.13338.

59. Noon MR, Eyre ELJ, Ellis M, Myers TD, Morris RO, Mundy PD, et al. The influence of recruitment age and anthropometric and physical characteristics on the development pathway of English academy football players. Int J Sports Physiol Perform. 2019;online first. doi:10.1123/ijspp.2019-0534.

60. O'Connor D, Larkin P, Mark Williams A. Talent identification and selection in elite youth football: an Australian context. Eur J Sport Sci. 2016;16:837–44. doi:10.1080/17461391.2016.1151945.

61. Roca A, Williams AM, Ford PR. Developmental activities and the acquisition of superior anticipation and decision making in soccer players. J Sports Sci. 2012;30:1643–52. doi:10.1080/02640414.2012.701761.

62. San ATL, Lee JLF. Developmental practice activities of elite youth swimmers. Movement, Health & Exercise. 2014;3:27–37. doi:10.15282/mohe.v3i0.15.

63. Sawai A, Mathis BJ, Natsui H, Zaboronok A, Mitsuhashi R, Warashina Y, et al. Risk of female athlete triad development in Japanese collegiate athletes is related to sport type and competitive level. Int J Womens Health. 2018;10:671–87. doi:10.2147/IJWH.S175446.

64. Sigmund P, Güllich A. Participation patterns of national-squad athletes in track-and-field and volleyball; 2020.

65. Smith SA. Retrospective practice histories of Division I and Divsion II female basketball players in the Carolinas [Doctoral Thesis]. Columbia: University of South Carolina; 2012.

66. Toering TT, Elferink-Gemser MT, Jordet G, Visscher C. Self-regulation and performance level of elite and non-elite youth soccer players. J Sports Sci. 2009;27:1509–17. doi:10.1080/02640410903369919.

67. Ureña CA. Skill acquisition in ballet dancers: the relationship between deliberate practice and expertise [Doctoral Thesis]. Tallahassee: Florida State University; 2004.

68. Ward P, Hodges NJ, Starkes JL, Williams MA. The road to excellence: deliberate practice and the development of expertise. High Ability Studies. 2007;18:119–53. doi:10.1080/13598130701709715.

69. Weissensteiner J, Abernethy B, Farrow D, Müller S. The development of anticipation: a cross-sectional examination of the practice experiences contributing to skill in cricket batting. J Sport Exerc Psychol. 2008;30:663–84. doi:10.1123/jsep.30.6.663.

70. Williams AM, Ward P, Bell-Walker J, Ford PR. Perceptual-cognitive expertise, practice history profiles and recall performance in soccer. Br J Psychol. 2012;103:393–411. doi:10.1111/j.2044-8295.2011.02081.x.

71. Young, BW, Salmela JH. Examination of practice activities related to the acquisition of elite performance in Canadian middle distance running. Int J Sport Psychol. 2010;41:73–90.

Supplementary Materials

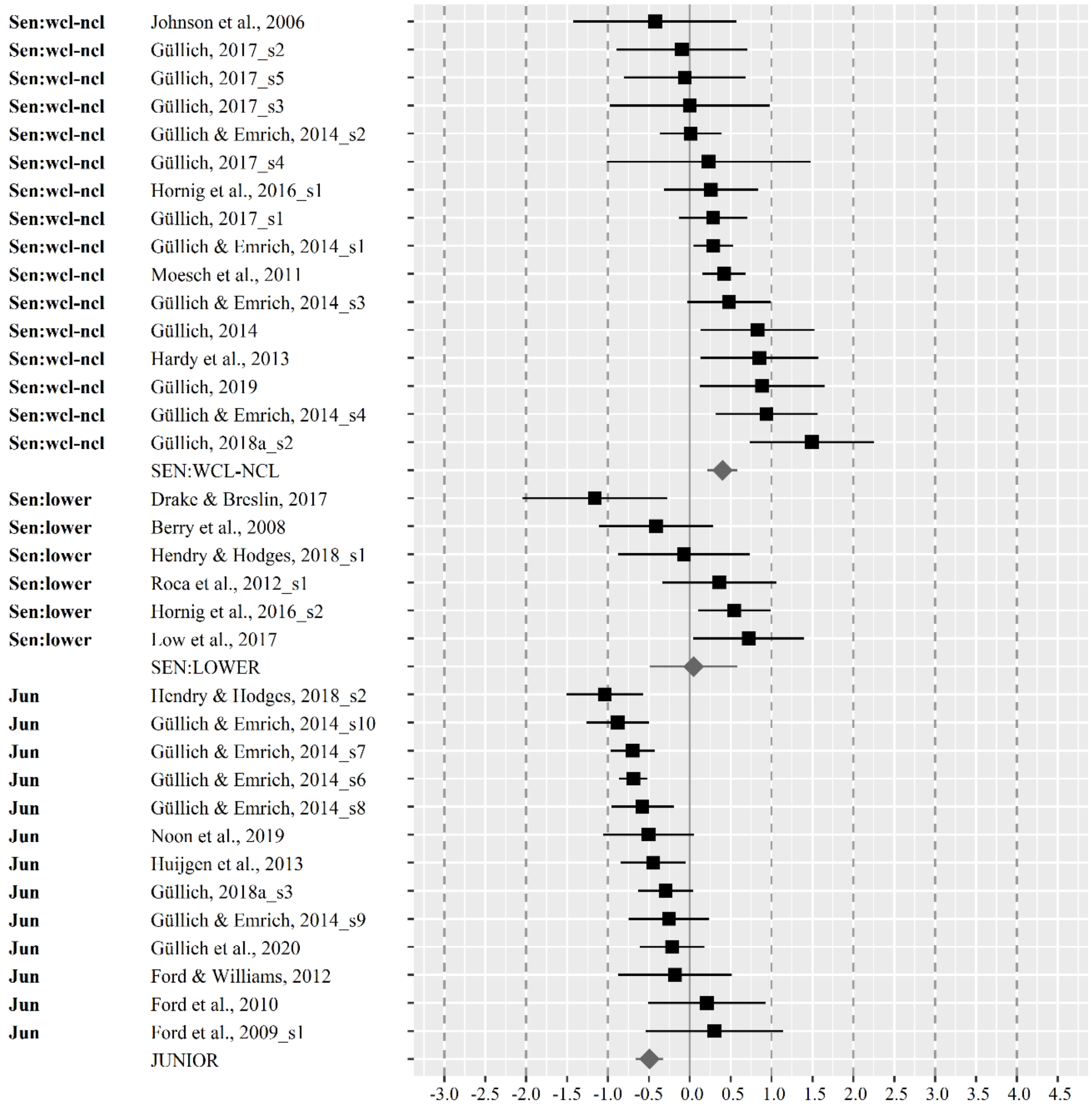

Figure S1. Age to reach performance milestones. Forest plot depicting each effect size (squares) and its 95% confidence interval (lines) entered into the meta-analytic model for differences between relatively higher and lower skilled athletes, along with the meta-analytic mean effect size overall and for junior athletes and senior athletes (diamonds). _s# refers to independent samples. Senior world-class vs. national-class athletes (Sen:wcl-ncl) I^2=39.82, Senior lower-level (Sen:lower) I^2=70.74, Junior (Jun) I^2=54.81.

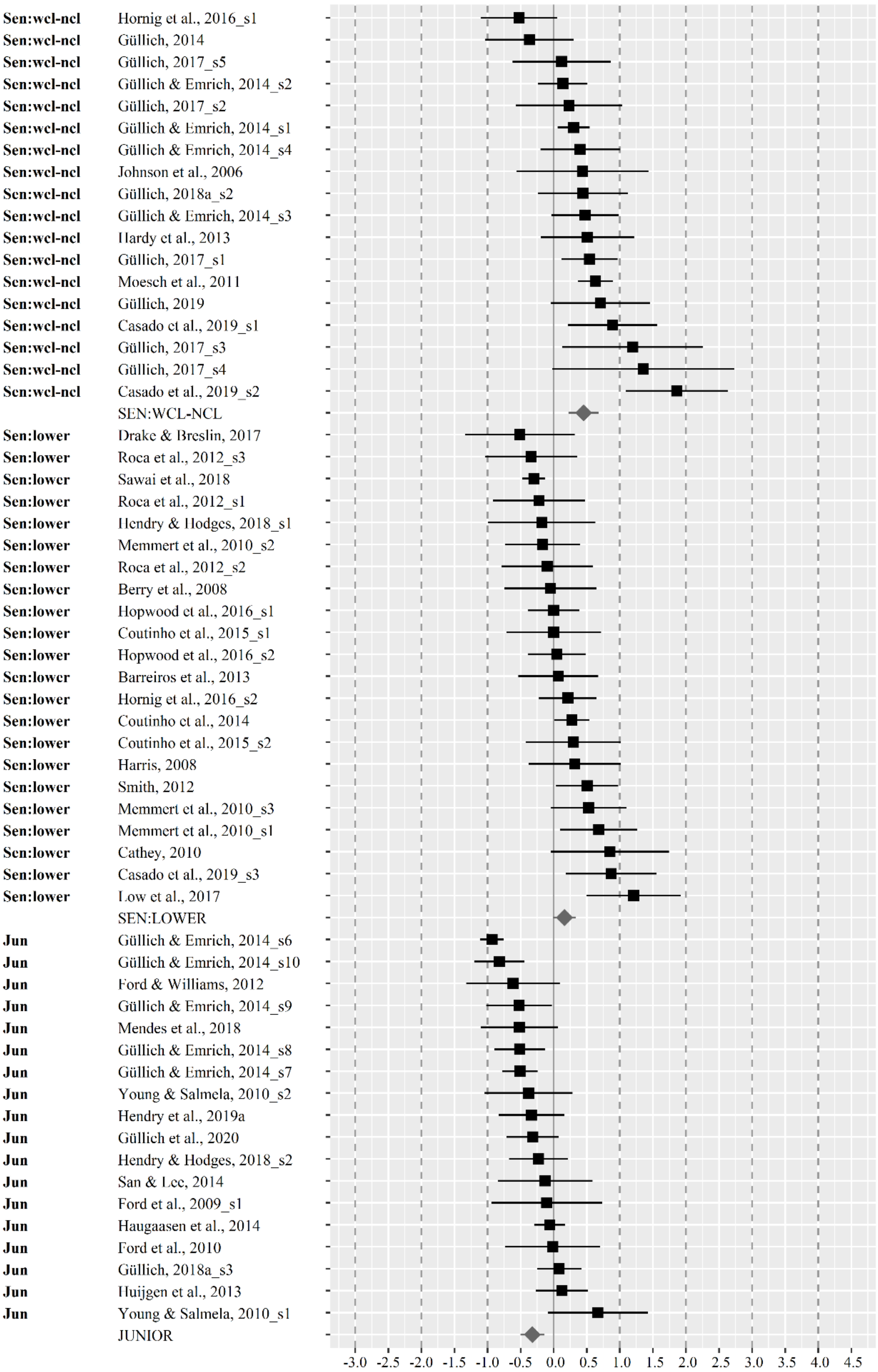

Figure S2. Main-sport starting age. Forest plot depicting each effect size (squares) and its 95% confidence interval (lines) entered into the meta-analytic model for differences between relatively higher and lower skilled athletes, along with the meta-analytic mean effect size overall and for junior athletes and senior athletes (diamonds). _s# refers to independent samples. Senior world-class vs. national-class athletes (Sen:wcl-ncl) I^2=50.48, Senior lower-level (Sen:lower) I^2=57.79, Junior (Jun) I^2=76.29.

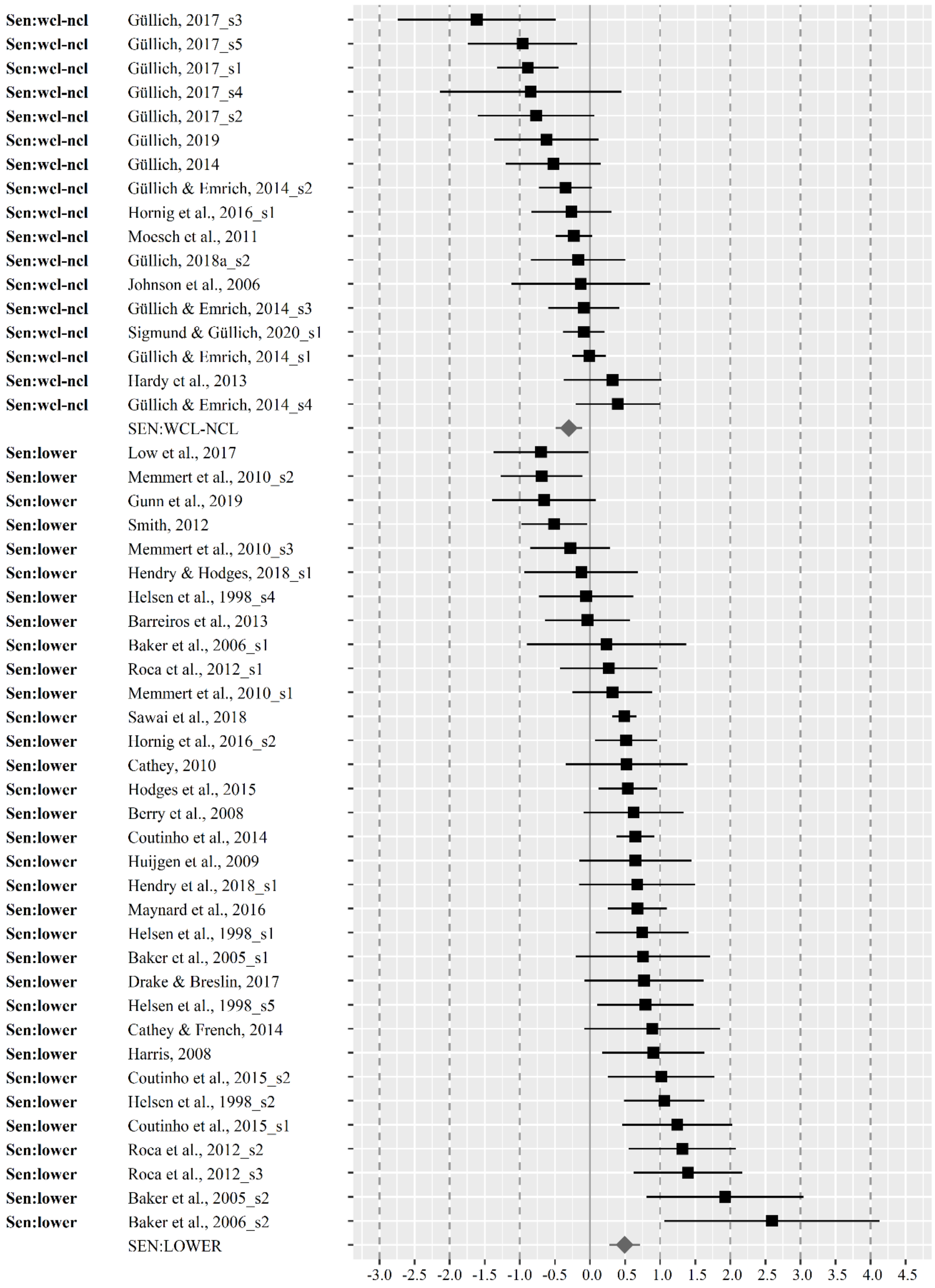

Figure S3a. Main-sport coach-led practice Seniors. Forest plot depicting each effect size (squares) and its 95% confidence interval (lines) entered into the meta-analytic model for differences between relatively higher and lower skilled athletes, along with the meta-analytic mean effect size overall and for junior athletes and senior athletes (diamonds). _s# refers to independent samples. Senior world-class vs. national-class athletes (Sen:wcl-ncl) I^2=32.33, Senior lower-level (Sen:lower) I^2=62.36.

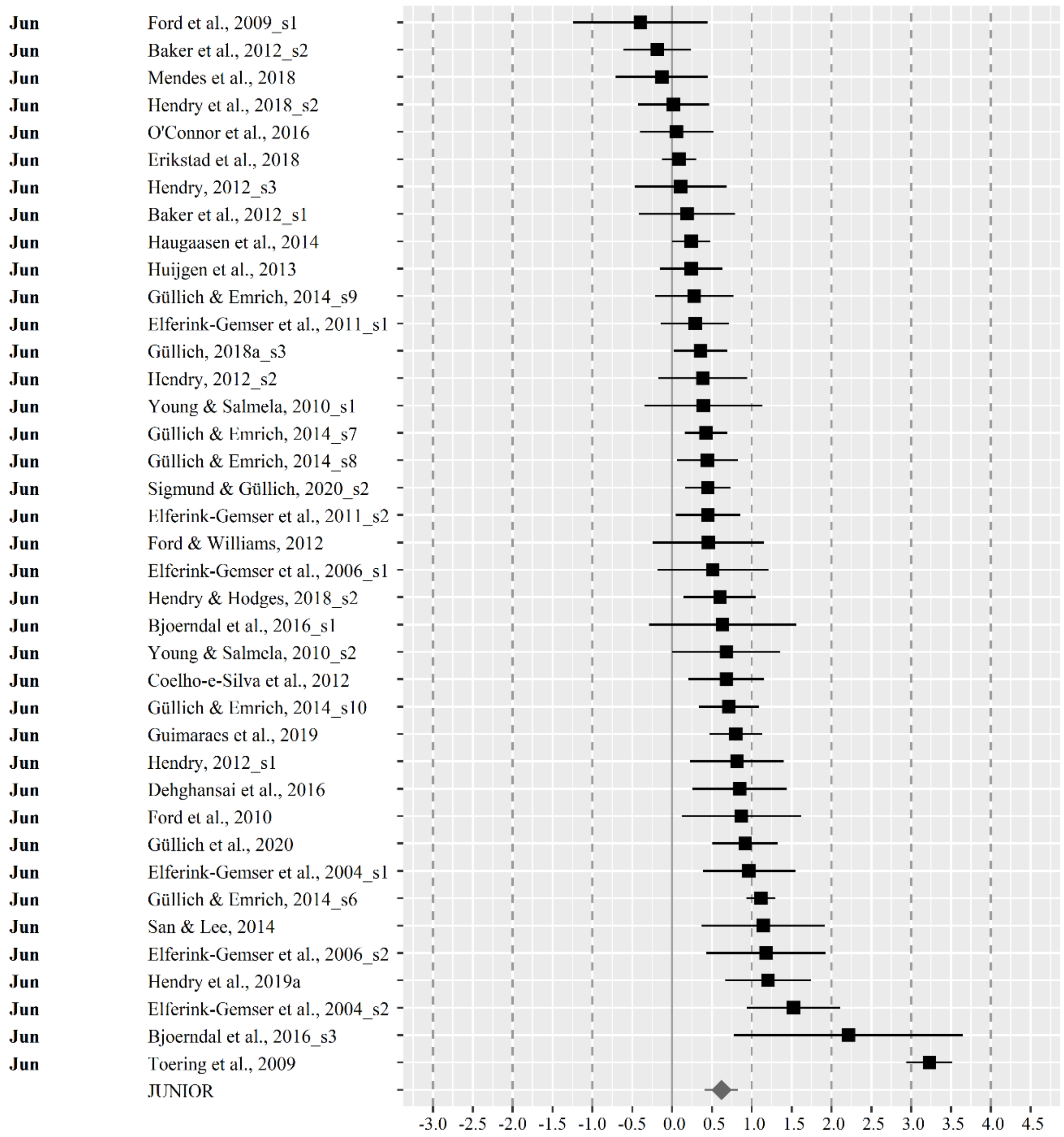

Figure S3b. Main-sport coach-led practice Juniors. Forest plot depicting each effect size (squares) and its 95% confidence interval (lines) entered into the meta-analytic model for differences between relatively higher and lower skilled athletes, along with the meta-analytic mean effect size overall and for junior athletes and senior athletes (diamonds). _s# refers to independent samples. Junior (Jun) I^2=91.79.

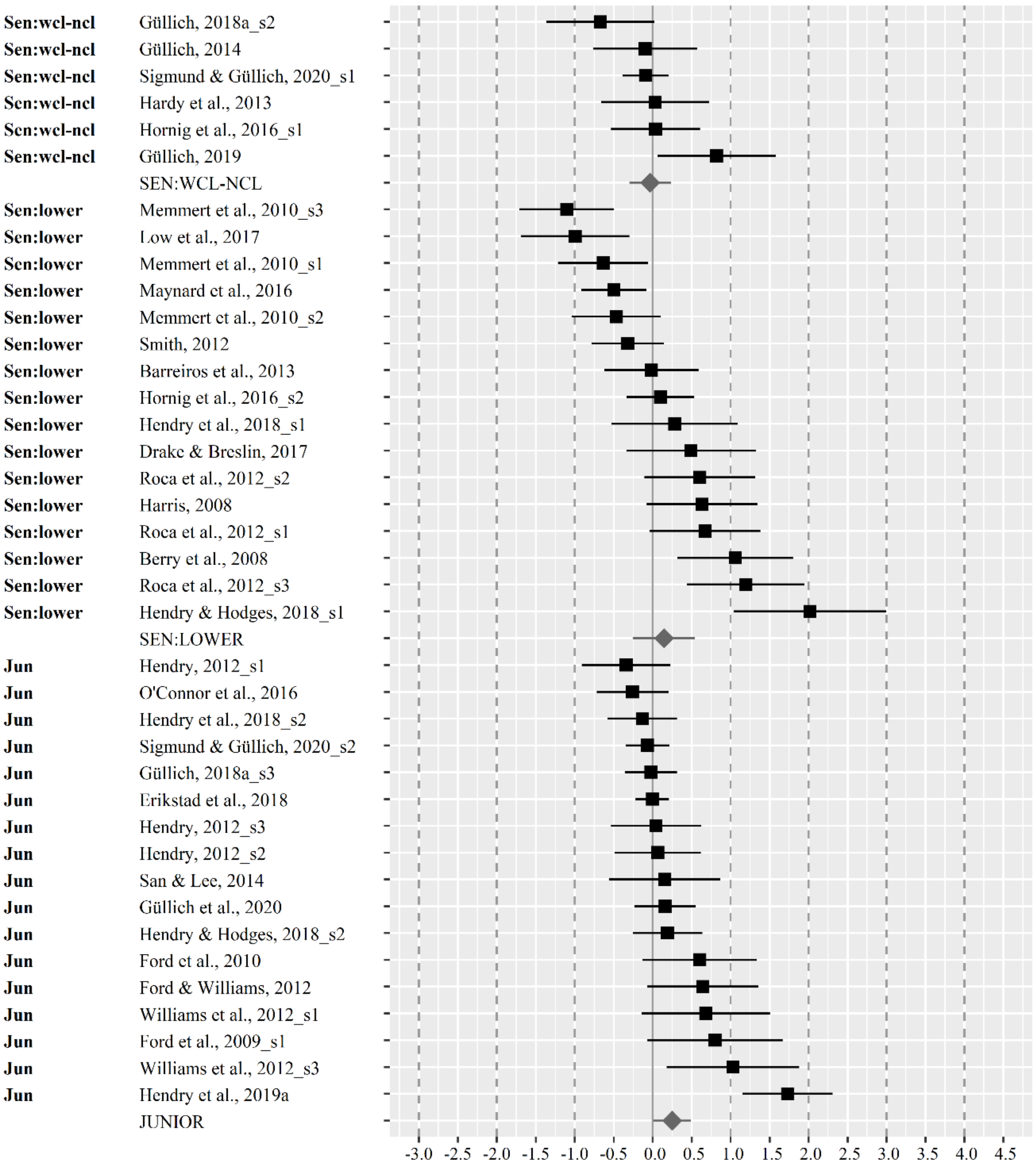

Figure S4. Main-sport peer-led play. Forest plot depicting each effect size (squares) and its 95% confidence interval (lines) entered into the meta-analytic model for differences between relatively higher and lower skilled athletes, along with the meta-analytic mean effect size overall and for junior athletes and senior athletes (diamonds). _s# refers to independent samples. Senior world-class vs. national-class athletes (Sen:wcl-ncl) I^2=40.39, Senior lower-level (Sen:lower) I^2=75.97, Junior (Jun) I^2=68.10.

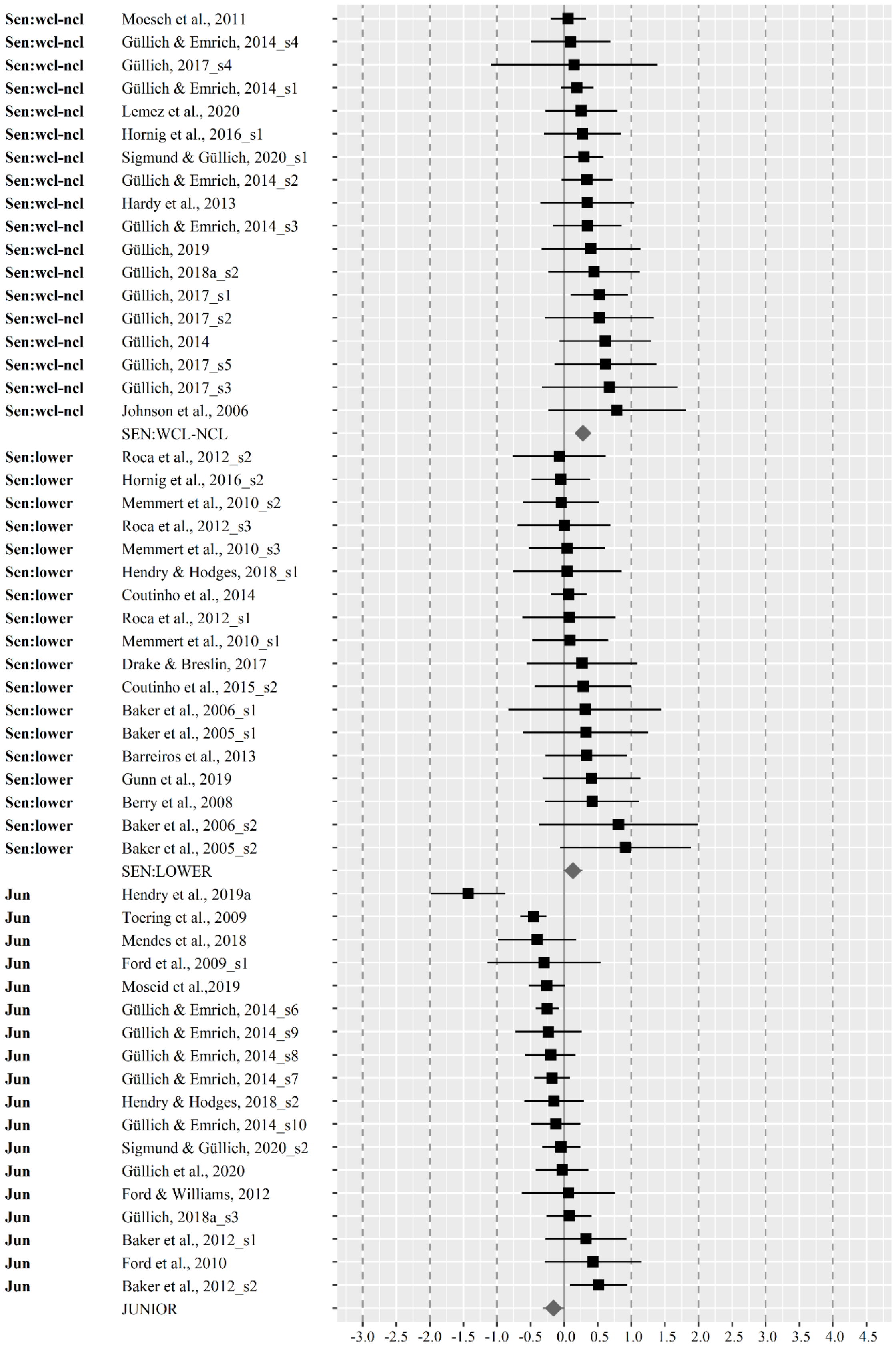

Figure S5. Other-sports practice and/or play. Forest plot depicting each effect size (squares) and its 95% confidence interval (lines) entered into the meta-analytic model for differences between relatively higher and lower skilled athletes, along with the meta-analytic mean effect size overall and for junior athletes and senior athletes (diamonds). _s# refers to independent samples. Senior world-class vs. national-class athletes (Sen:wcl-ncl) I^2=0.0, Senior lower-level (Sen:lower) I^2=0.0, Junior (Jun) I^2=64.81.

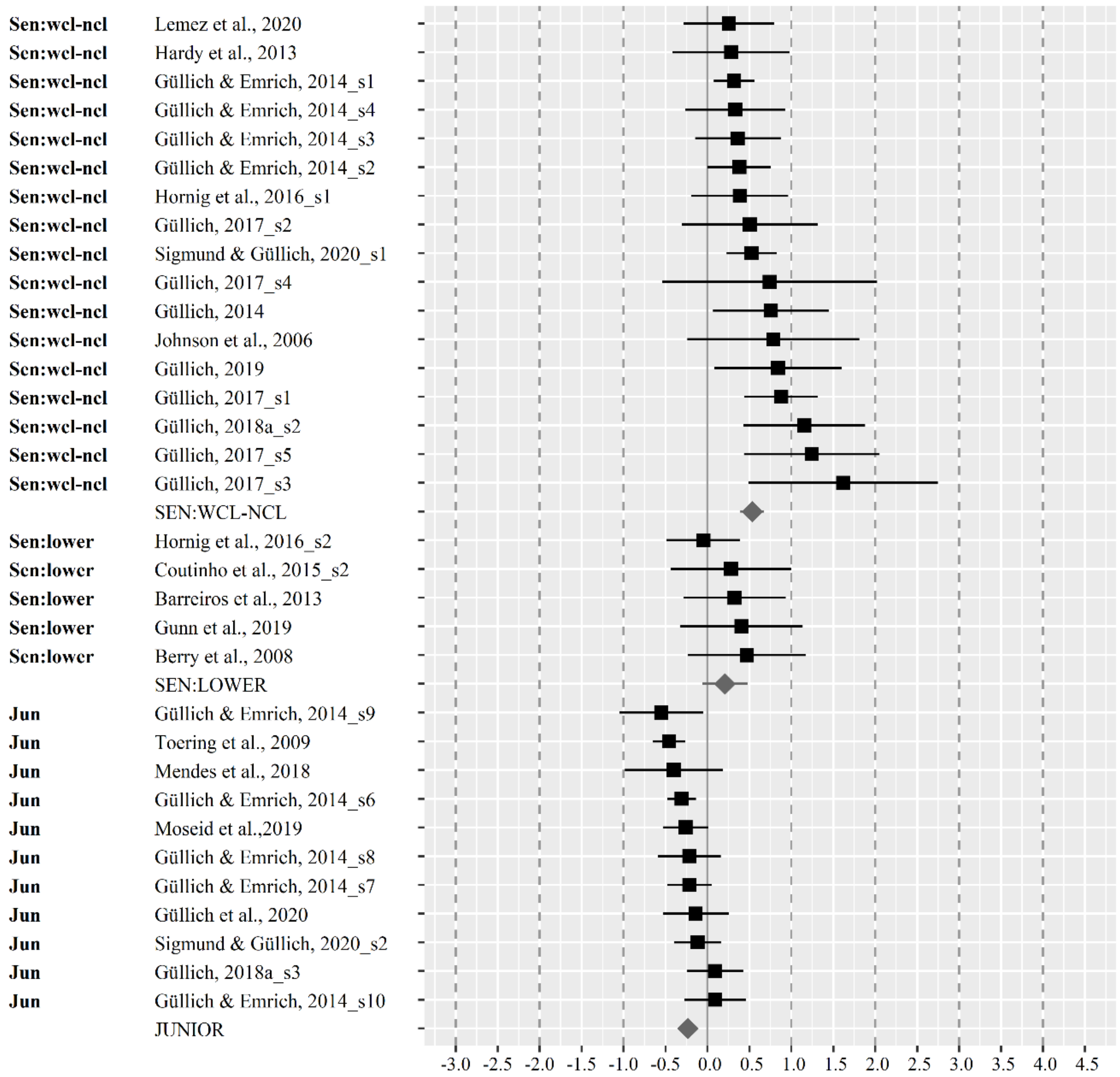

Figure S6. Other-sports practice. Forest plot depicting each effect size (squares) and its 95% confidence interval (lines) entered into the meta-analytic model for differences between relatively higher and lower skilled athletes, along with the meta-analytic mean effect size overall and for junior athletes and senior athletes (diamonds). _s# refers to independent samples. Senior world-class vs. national-class athletes (Sen:wcl-ncl) I^2=0.0, Senior lower-level (Sen:lower) I^2=0.0, Junior (Jun) I^2=33.69.

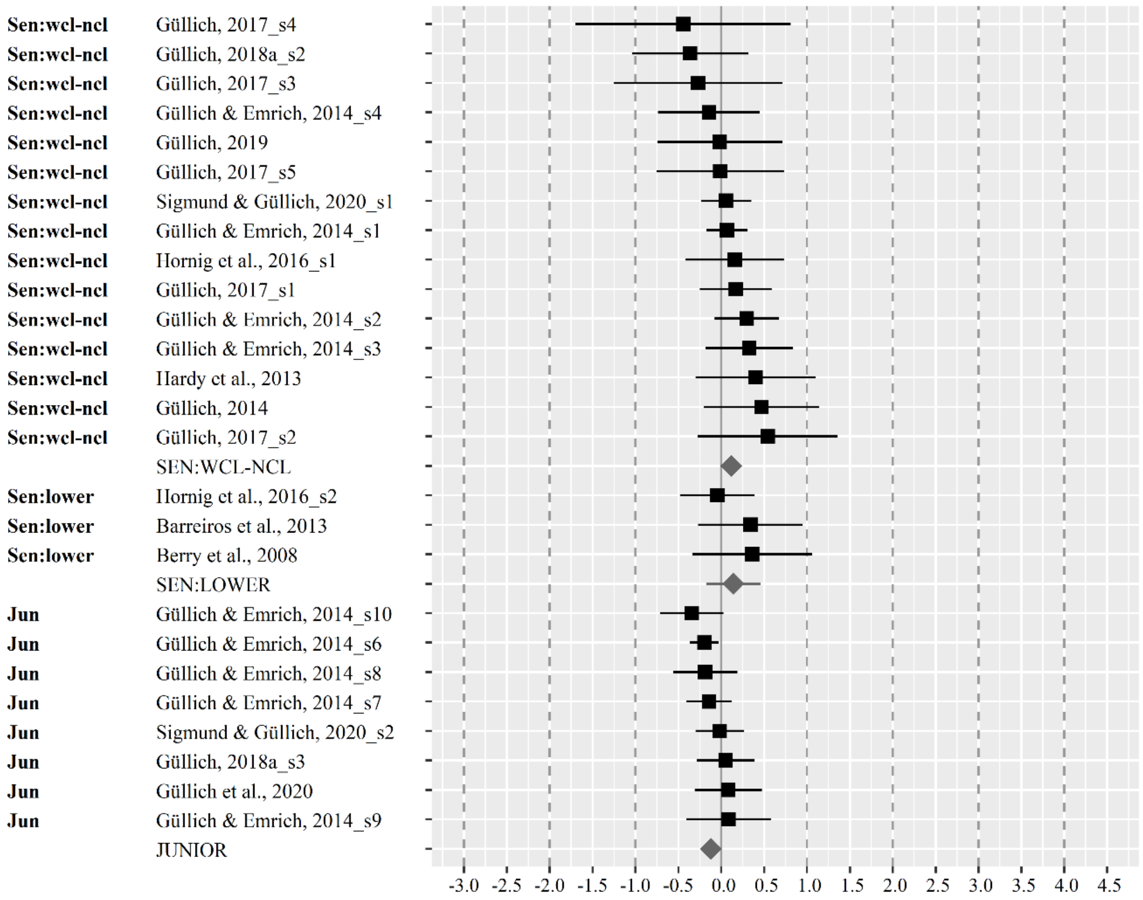

Figure S7. Other-sports play. Forest plot depicting each effect size (squares) and its 95% confidence interval (lines) entered into the meta-analytic model for differences between relatively higher and lower skilled athletes, along with the meta-analytic mean effect size overall and for junior athletes and senior athletes (diamonds). _s# refers to independent samples. Senior world-class vs. national-class athletes (Sen:wcl-ncl) I^2=0.0, Senior lower-level (Sen:lower) I^2=0.0, Junior (Jun) I^2=0.0.

Publication bias analyses

Publication bias occurs when certain types of effects, such as small effects, null effects, or effects that are in the opposite direction to researchers' expectations, are systematically suppressed from publication.

We first examined the funnel plots for each overall model for asymmetry. Funnel plots depict the relationship between standard error and effect size. The smaller the study's standard error, the closer that study's effect size should be to the mean effect. The larger the study's standard error, the more random error it has. Due to its random nature, the study's effect size should have an equally likely chance of being higher or lower than the mean effect size. If a funnel plot is asymmetrical, i. e., if smaller studies (with larger standard error) are "missing" from one side of the plot, those studies might be missing due to publication bias. We also quantified symmetry with Egger's regression (Egger et al., 1997). However, Egger's regression is prone to Type I error when heterogeneity is high (Sterne et al., 2011), as is the case in these meta-analytic models. Thus, Egger's regression results should be interpreted with caution.

As can be seen in Figure S8, the funnel plots are by and large symmetrical. Egger's regression supports this observation for age to reach milestones, main-sport practice, main-sport play, and other-sports practice and/or play. However, Egger's regression was significant, suggesting there could be publication bias, in starting age, other-sports practice, and other-sports play ($.003 < p < .031$). The analyses suggest that associations of higher performance with a *later* starting age or *more* other-sports practice or play in small samples may have been more likely to be published,

We next examined a potential moderator effect of publication status, i. e., whether the mean effect sizes differed between published and unpublished studies. Significant results would suggest that larger (or smaller) effects are more likely to be published. There was no evidence for differences in effect size by publication status (i. e., there was no significant moderation effect of publication status) for any of the predictor variables when considering the entire sample, only juniors, or only seniors ($0.00 < F < 2.45$, $.126 < p < .967$). The results suggest that smaller or larger than average effects were not less likely to be published in this literature.

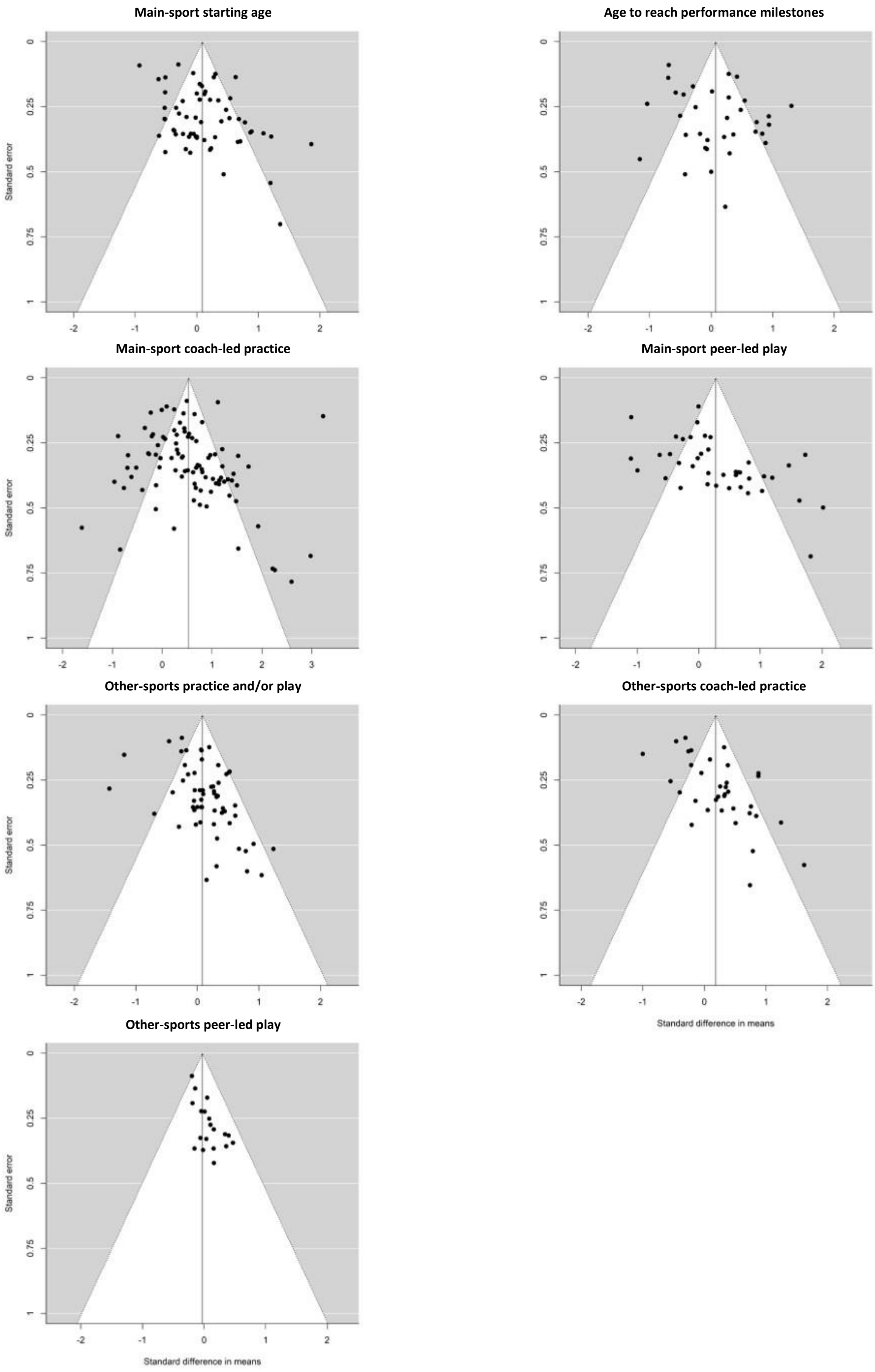

Figure S8. Funnel plots for the seven predictors.

Table S2. GRADE rating of the quality of evidence.

	**Study design**(a)	**Consistency**	**Directness**	**Precision**	**Quality of evidence**(a)
**Age of milestones**					
Junior performance	moderate	high	high	high	moderate
Senior performance	moderate	high	high	high	moderate
Senior WCl vs. NCl	moderate to high(b)	high	high	high	moderate to high
Junior vs. senior	moderate	high	high	high	moderate
**Starting age**					
Junior performance	moderate	high	high	high	moderate
Senior performance	moderate	high	high	high	moderate
Senior WCl vs. NCl	moderate to high(b)	high	high	high	moderate to high
Junior vs. senior	moderate	high	high	high	moderate
**Main-sport practice**					
Junior performance	moderate	high	high	high	moderate
Senior performance	moderate	high(c)	high	high	moderate
Senior WCl vs. NCl	moderate to high(b)	high	high	high	moderate to high
Junior vs. senior	moderate	high	high	high	moderate
**Main-sport play**					
Junior performance	low	low	high	high	low
Senior performance	low	low	high	high	low
Senior WCl vs. NCl	low	low	high	moderate	low
Junior vs. senior	low	low	high	high	low
**Other-sports practice**					
Junior performance	moderate	high	high	high	moderate
Senior performance	moderate	high	high	high	moderate
Senior WCl vs. NCl	moderate to high(b)	high	high	high	moderate to high
Junior vs. senior	moderate	high	high	high	moderate
**Other-sports play**					
Junior performance	low	low	high	high(d)	low
Senior performance	low	low	high	high	low
Senior WCl vs. NCl	low to moderate(b)	low	high	high	low
Junior vs. senior	low	low	high	high(d)	low

(a) Dose-response gradient for all predictor variables except main-sport play and other-sports play. (b) Several matched-pairs studies with consistent findings. (c) When only considering effect of *early* main-sport practice (up to age 15 years) on senior performance, consistency is low. (d) When only considering effect of *early* other-sports play (up to age 15 years) on junior performance, precision is low.
